# Advancements and Challenges in the Management of Prosthetic Valve Endocarditis: A Review

**DOI:** 10.3390/pathogens13121039

**Published:** 2024-11-26

**Authors:** Francesco Nappi

**Affiliations:** Department of Cardiac Surgery, Centre Cardiologique du Nord, 93200 Saint-Denis, France; francesconappi2@gmail.com or f.nappi@ccn.fr; Tel.: +33-149334104; Fax: +33-149334119

**Keywords:** infective endocarditis, prosthetic valve endocarditis, TAVR, vegetation

## Abstract

Prosthetic valve endocarditis (PVE) is the medical term used to describe a focus of infection involving a valvular substitute within the heart. It is a significant concern in the field of cardiology, and the epidemiology of PVE has seen notable developments over the last five decades. The disease currently affects an older demographic and is becoming increasingly prevalent in patients with transcatheter-implanted valves. It is imperative that we urgently address the significant challenges posed by PVE. It is a disease that has a wide range of potential aetiologies, clinical presentations, and courses. In developed countries, *Staphylococcus aureus* is now the predominant causative organism, resulting in an aggressive form of disease that frequently afflicts vulnerable or elderly populations. However, it is clear that *Enterococcus* species present a significant challenge in the context of PVE following TAVR procedures, given their elevated prevalence. The 2023 Duke/International Society for Cardiovascular Infectious Diseases infective endocarditis diagnostic criteria now include significant developments in microbiological and image-based techniques for diagnostic purposes, specifically the incorporation of fluorine-18 fluorodeoxyglucose positron emission tomography and computed tomography. These developments unequivocally enhance the diagnostic sensitivity for PVE, while maintaining the specificity. They do so in accordance with the results of studies conducted specifically for the purpose of validation. The lack of rigorous scientific studies and a shortage of funding and resources for research have led to a significant gap in our understanding. Randomized controlled trials could provide invaluable insight and guidance for clinical practice, but they are missing, which represents a major gap. It is clear that there is an urgent need for more research. PVE is a life-threatening condition that must be handled by a multidisciplinary endocarditis team at a cardiac centre in order to improve outcomes. The emergence of innovative surgical techniques has empowered clinicians to steer more patients away from surgical procedures, despite the presence of clear indications for them. A select group of patients can now complete parenteral or oral antimicrobial treatment at home. Additionally, antibiotic prophylaxis is the best option for individuals with prosthetic valves who are going to have invasive dental procedures. These individuals should be given antibiotics beforehand.

## 1. Introduction

Over half a century has elapsed since the initial reports of prosthetic valve endocarditis (PVE). Since that time, there have been a number of significant advances in the understanding and management of PVE. These include developments in epidemiology [1,2,3], with the ageing of the population and the introduction of transcatheter valve therapies [4,5,6], as well as an enhanced role for advances in molecular microbiological laboratory methods and nuclear medicine diagnostic techniques. Other notable advances include the formation of structured multispecialty endocarditis teams [7,8], new surgical procedures, and a structured cohort of individuals who have elected to forego surgery, despite presenting with indications for surgical intervention. It is now feasible to complete antimicrobial treatment in the patient’s home, either intravenously or orally, which represents an important advance [9,10]. In addition, there have been significant developments in prevention [7,8,11]. These developments justify an update to the current guidelines, which address prosthetic valve endocarditis.

## 2. Epidemiology: Searching for New Points in PVE Patients—TAVR vs. SAVR?

**Figure 1:**
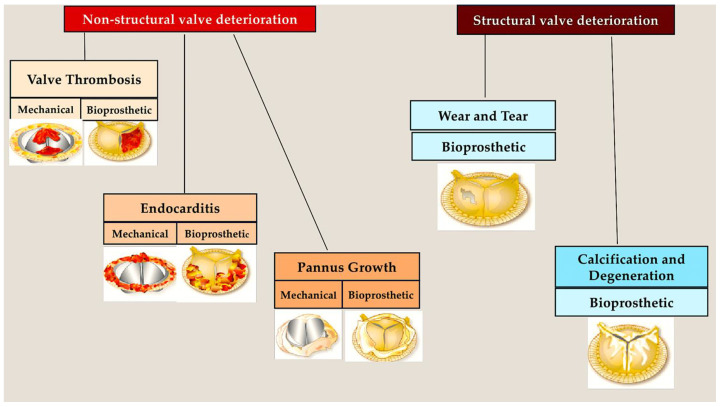
Illustration demonstrates the various pathophysiological processes (thrombosis, endocarditis, pannus growth, wear and tear, calcification, and deterioration) that can occur in mechanical and bioprosthetic valves, leading to both structural and nonstructural valve deterioration.

Initial research indicated that PVE constituted 5% of all infective endocarditis (IE) cases [12,13,14]. However, recent evidence suggests that this prevalence has increased to 20% over the past few decades [15,16]. The incidence of IE in patients who have undergone the surgical implantation of prosthetic valves is reported to vary from 0.5 to 1.0 per 100 person-years. This incidence is elevated in patients who have received biologic valve implants than for those who have been fitted with mechanical valves [16,17,18,19,20,21] (Figure 1).

In certain high-income countries, such as the United States, there has been a significant increase in the number of people who inject drugs, leading to a corresponding rise in PVE cases. This has resulted in a higher likelihood of developing complex PVE, which, in turn, has led to a rise in mortality rates [22,23,24].

The incidence of IE following transcatheter aortic valve replacement (TAVR) has been reported to range from 0.3 to 2.0 per 100 person-years [4,25]. The results of a meta-analysis of randomised controlled trials comparing TAVR with surgical aortic valve replacement revealed no statistically significant discrepancies in the incidence of in-hospital, delayed, and cumulative infective endocarditis between the two populations of patients [26]. One might posit that TAVR would result in a reduced incidence of early IE due to its less invasive nature. However, the extant evidence does not substantiate this hypothesis [27]. Notably, TAVR is associated with a sixfold increased risk of early periprocedural IE (within 100 days), with the majority of incidents (64%) documented during the first 12 months following the procedure [28]. One potential rationale for this inconsistency is the elevated prevalence of comorbidities seen in individuals who have undergone TAVR, with a particularly elevated incidence occurring immediately following the TAVR implantation procedure. Those patients with a high comorbidity index, including those who frequently require care within healthcare settings, for example, those undergoing dialysis, are at a heightened risk of contracting healthcare-associated infections. It is reasonable to hypothesise that this variable may play a contributory role in the unusual pattern observed in IE incidence rates among patients who have proceeded with TAVR.

The occurrence of hospital-acquired infections (HAIs) among patients with prosthetic valves is a well-documented phenomenon, particularly among those who have undergone the placement of a prosthetic valve. The underlying comorbidity of these patients, which was previously discussed, contributes to the elevated risk for HAIs. It is clear that over half of TAVR IE cases can be classified as healthcare-associated, representing a rate that is more than double that observed in the IE cases of surgical aortic valve replacement (SAVR) [4]. It is evident from the currently available evidence on the outcomes of transcatheter valvular procedures other than TAVR in patients with IE that no meaningful conclusions can be drawn.

### 2.1. The Causative Pathogens

**Figure 2:**
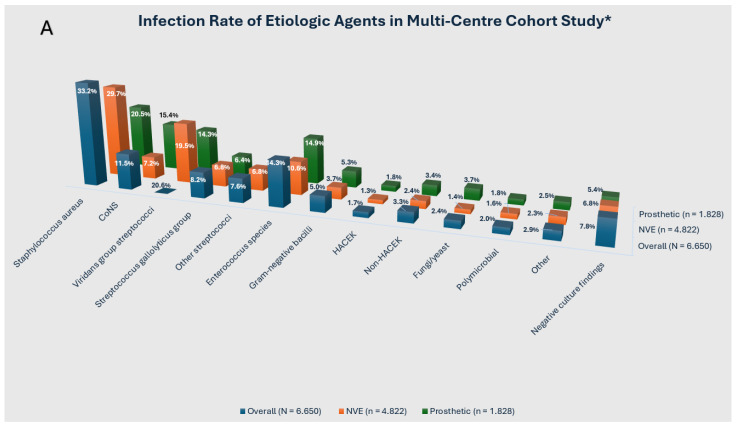

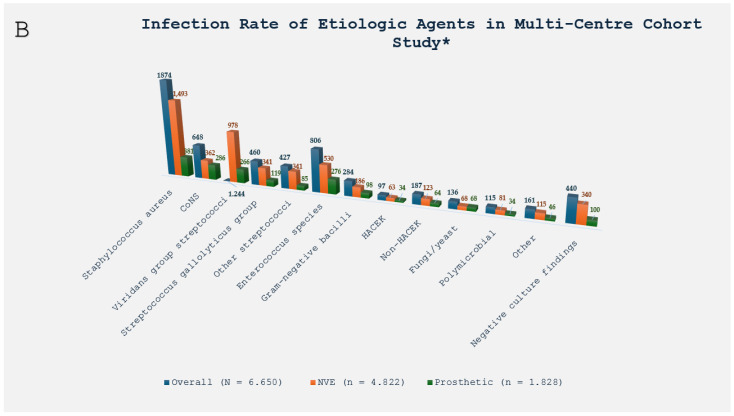
The illustration depicts the prevalence of etiological causative microorganisms documented in a multicentre cohort of the ICE *, as indicated by both the percentage of cases (**A**) and the number of cases (**B**). The columns representing the overall population, the NVE group, and the PVE group are coloured blue, orange, and green, respectively. Abbreviations: CoNS, coagulase-negative staphylococci; HACEK, *Haemophilus* species, *Aggregatibacter actino mycetemcomitans*, *Aggregatibacter aphrophilus* (previously, *Haemophilus aphrophilus* and *Haemophilus paraphrophilus*), *Cardiobacterium hominis*, *Eikenella corrodens*, and *Kingella* species; * ICE, International Collaboration on Endocarditis; NVE, native valve endocarditis; PVE, prosthetic valve endocarditis [29,30,31].

A marked disparity has been observed between the microbiological profiles of PVE patients, occurring at approximately the 12-month postoperative mark, and those of late PVE cases, occurring beyond the 12-month postoperative threshold. In the context of early infection, it has been reported that cases acquired in a healthcare setting, such as in hospitals or other medical facilities, constitute the majority of instances, occurring mainly within the first two months after a surgical procedure. The majority of these acquisitions are identified as *Staphylococcus epidermidis*, exhibiting resistance to methicillin, and *Staphylococcus aureus*. *Enterococci*, Gram-negative rods (including *Enterobacter* spp., *Serratia* spp., and *Pseudomonas* spp.), and *Candida* spp. are also identified, albeit with less frequency, as the pathogens involved [1,29,30,31,32] (Figure 2A,B).

**Figure 3:**
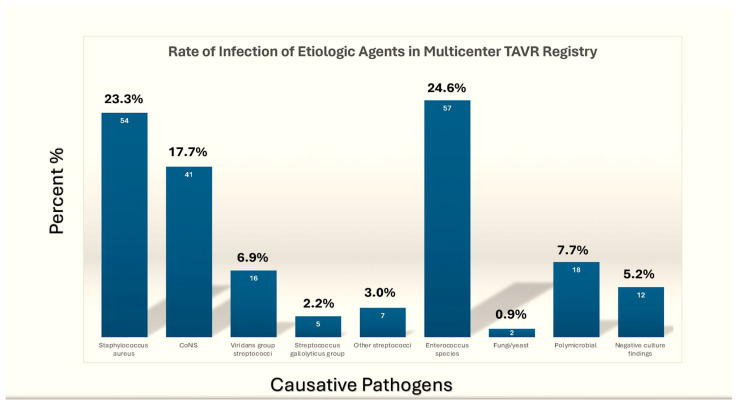
Illustration of the documented prevalence of etiological causative pathogens in a multicentre cohort study of the TVAR. Abbreviations: CoNS, coagulase-negative staphylococci; HACEK, *Haemophilus* species, *Aggregatibacter actino mycetemcomitans*, *Aggregatibacter aphrophilus* (formerly, *Haemophilus aphrophilus* and *Haemophilus paraphrophilus*), *Cardiobacterium hominis*, *Eikenella corrodens*, and *Kingella* species; values are n (patients). International TAVR Registry [30].

Late PVE occurring >12 months is most commonly a healthcare-associated infection, with a microbial profile similar to that observed in NVE. Nevertheless, it is probable that this progression from the initial, nosocomial phase and the subsequent, late community phase will occur earlier than the first year following valve insertion, a hypothesis that some investigators have proposed [25]. It is also pertinent to consider the potential for nosocomial (and even intraoperative)-acquired PVE, which may manifest clinically at a later stage as a result of *Cutibacterium acnes*, a species of low-virulent anaerobic bacteria that is a common cause of infections in indwelling devices [33]. TAVR IE has the potential to facilitate the transmission of healthcare-associated infections [30]. In a number of cases, the predominant causative agents have been *staphylococcus aureus* and, notably, *enterococcus* [4]. The latter can account for a significant proportion of the causative pathogens, reaching approximately 30% in some studies, which is a cause for concern. The high prevalence of early infection by enterococci has been attributed to a number of factors, with the predominant hypothesis being the widespread utilisation of the femoral access approach for TAVR [34,35,36,37,38,39,40] (Figure 3).

### 2.2. How Metagenomics Helps Diagnose Microbiology

The utilisation of molecular methodologies, encompassing polymerase chain reaction (PCR) and metagenomic next-generation sequencing, has proven to be of immense value in the etiological diagnostic evaluation of PVE through the examination of resected valvular prostheses [41,42]. Furthermore, the available evidence indicates that the examination of blood samples may prove to be an additional, beneficial measure. A recently published study from a reference centre presents the findings of a preliminary prospective evaluation of the efficacy of a PCR targeting the V1 to V3 domain of the 16S ribosomal RNA gene (sequenced using next-generation Illumina technology). The genetic composition of infective endocarditis pathogenic organisms was identified in 70% of cases, even in instances where culture-based diagnostics yielded nondetectable results. Of these subjects, 39%, or 8 out of the 21 subjects, had been diagnosed with PVE, and 63% had been diagnosed with IE. The samples were taken from blood and plasma [43]. With respect to the identification of circulating bacterial DNA in plasma, the extant evidence is comparatively scarce [44].

## 3. Assessing Risk Factors

The evidence derived from nonrandomised studies indicates that mechanical prostheses and the Ross procedure are more effective than bioprosthetic valves in preventing the development of PVE over the long term. In a propensity-score-matched cohort of patients younger than 50 years of age and without any concomitant comorbidities, the Ross procedure was linked to a reduced incidence of PVE than surgical bioprosthesis aortic valve replacement (hazard ratio [HR]: 0.37; 95% confidence interval [CI]: 0.17–0.80; *p* = 0.012). Indeed, at the 15-year point in time, the cumulative incidence of PVE in the Ross cohort reached 2.3% (95% CI: 1.1–4.3%), as evidenced by the data [45].

### 3.1. Ross Technique vs. Other Valve Types

A significant corpus of evidence from propensity-matched observational studies and meta-analysis has demonstrated that Ross surgery is correlated with extended survival into the second postoperative decade [46,47,48,49,50,51,52,53,54,55,56,57]. This finding is consistent across numerous studies [46,48,51,54,55,58,59,60,61,62,63,64,65,66,67,68,69,70,71,72,73,74,75,76,77,78,79,80,81,82,83,84,85,86,87,88,89,90,91,92]. The majority of these studies report similar survival rates to the age- and sex-matched general population, indicating that the observed survival advantage is not merely a consequence of the demographic characteristics of the study population. The aforementioned series of patients who have undergone the Ross procedure demonstrate superior results compared with other published studies on aortic valve surgery in younger and middle-aged adults. These results are particularly notable in terms of survival rates, which are comparable with those observed in a matched general population. Additionally, studies evaluating specific, well-defined cohorts of patients who underwent conventional AVR also reported encouraging outcomes [93,94,95,96,97,98].

Similarly, a significant body of research demonstrates the superior efficacy of the Ross procedure compared with alternative techniques. A notable number of the aforementioned reports employed a comparative methodology, contrasting the observed survival rates of the study cohort at the 10-year follow-up with those of a demographically matched general population. This methodology was utilized in a multitude of reports [45,47,48,49,50,51,52,53,54,56,58,93,94,96,98]. In these instances, the comparative analysis yielded a positive outcome, particularly in instances where patients who underwent the AVR procedure received a pulmonary autograft and those who underwent pulmonary autograft surgery with conventional bioprosthetic valves [93,94,98,99]. Furthermore, five cohort studies demonstrated that the Ross procedure was associated with enhanced long-term clinical outcomes during the second postoperative decade [45,47,50,51,52,53,54].

In opposition to the aforementioned findings, three studies [31,32,36,46] have indicated that both mechanical and bioprosthetic valves are associated with marginally elevated long-term mortality rates when implanted in young and middle-aged adults, in comparison with the corresponding general population. It should be highlighted that the most favourable outcomes have been reported in a large series of Ross procedures carried out in experienced institutions, with long-term survival ranging from 87% to 95% at 15 years. The incidence of Ross-related reoperations has exhibited a greater range of variability, with rates oscillating between 75% and 94% after a 15-year follow-up period [45,47,48,50,51,52,53,54,58,100]. It is noteworthy that the preponderance of patients who received a Ross procedure in the extensive series were aged between 34 and 44 years, giving rise to a reoperation rate of only 1% to 2% per patient-year. This low reoperation rate is highly encouraging and contrasts with that observed with conventional stented bioprosthetic valves. It is noteworthy that Takkenberg and colleagues [100] observed that the Ross procedure exhibited a low incidence of long-term valve-related complications. The meta-analysis of observational studies revealed that the linearised failure rates of pulmonary autografts and pulmonary homograft structural valve deterioration were relatively low (0.78% and 0.55% per patient-year, respectively). The incidence of endocarditis associated with the use of pulmonary autograft and pulmonary homograft valve was found to be 0.26% and 0.20% per patient-year, respectively. The incidence of thromboembolism, bleeding, or valve thrombosis was 0.36% per patient-year. To the best of our knowledge, there are no randomised trials that have evaluated the Ross procedure in comparison with bioprosthetic AVR in patients with severe aortic valve disease (S-AVD). Nevertheless, a recent patient-level meta-analysis provides compelling evidence that the Ross procedure is more effective than other approaches [57].

The evidence currently available supports the use of the Ross procedure, as it provides an added survival advantage over mechanical aortic valve replacement. A propensity-matched study was conducted four years ago, which included a cohort of 416 young and middle-aged adults. A follow-up period of over 14 years revealed a statistically significant reduction in the hazard ratio (HR) for cardiac and valve-related mortality in patients who underwent the Ross procedure (n = 208) compared with those who received conventional mechanical AVR (n = 208) (97% vs. 89% at 20 years; hazard ratio: 0.22; *p* = 0.03) [101]. The investigation revealed no statistically notable discrepancy in the early outcomes and overall survival rates when comparing the two groups. Furthermore, the proportion of patients who were free from the need for repeat procedures at the 20-year mark was similar in both groups (87% in the Ross group vs. 94% in the mechanical AVR group; hazard ratio: 1.86; *p* = 0.19). It should be noted that 43% of patients who received the Ross surgery had aortic insufficiency prior to the procedure. Reoperations in this group included any subsequent surgical or percutaneous reintervention at the aortic and/or pulmonary position(s). In comparison with alternative surgical techniques, the Ross procedure has been demonstrated to result in a significantly lower incidence of thromboembolic and major haemorrhagic complications over the longer term, with a success rate of 99% at 20 years. In comparison, the success rate for alternative procedures is 80%. The calculated hazard ratio was found to be 0.09, with a *p*-value < 0.001 [55].

A study was conducted by Buratto and colleagues on a total of 1928 patients who underwent isolated mechanical AVR and 392 patients who underwent the Ross procedure. A risk-adjusted analysis was performed on the study, which also had a follow-up period exceeding 25 years. A substantial improvement in mortality was observed with the Ross procedure in 275 propensity-score-matched pairs (94% vs. 84%; *p* = 0.018), whilst 30-day mortality was similar (Ross 0%; mechanical AVR 0.4%; *p* > 0.99) [101]. It has yet to be demonstrated by any study with a large propensity-matched analysis that patients who have undergone the Ross procedure have greater freedom from mortality of all causes compared with those who have undergone mechanical AVR. It can be proposed that a potential advantage associated with the Ross procedure may render the Ross reversal procedure a favourable option [102]. This strategy has been demonstrated to be carried out with a low incidence of complications and satisfactory pulmonary valve functionality, which can mitigate the risk of the patient losing two native valves in the event of autograft failure in the aortic position. No additional postoperative complications of consequence were identified, and the median length of hospitalisation following surgery was 7.2 days (range: 4–41 days) [102].

### 3.2. What Is the Gold Standard for Prosthetic Valve Endocarditis (PVEs) and What Is the Pathophysiology?

There are few randomized surgical studies examining PVE incidence by category of previously used prosthesis. Nevertheless, the available evidence from direct observation indicates that mechanical substitutes demonstrate greater long-term durability against PVE than bioprosthetic valves. This approach has been put forth as a consequence of enhanced valve performance and the prevention of structural deterioration of the valve, which appears to be a predisposing factor for infection. The markedly reduced prevalence of IE following the Ross procedure reinforces the hypothesis that the utilization of prosthetic valves in lieu of autologous tissue (i.e., pulmonary autograft) constitutes a risk factor for the onset of prosthetic valves endocarditis, even in the absence of underlying cardiac pathology [55,103,104].

The occurrence of high-velocity streams of blood flow, in conjunction with the observed impairment in bioprosthetic leaflet movement in cases of degeneration, may result in the uninhibited aggregation of platelets and blood (a form of nonbacterial thrombotic endocarditis). This, in turn, may foster the creation of a milieu that is conducive to subsequent adhesion and the perpetuation of bacterial organisms. The question of whether surgical aortic valve replacement with bioprosthetic valves entails a distinct probability of postprocedural PVE compared with TAVR persists without a definitive answer.

Nevertheless, the latest results of the ongoing follow-up studies, which have been monitored for up to 9 years [105,106], indicate that the proportion of patients who develop PVE is similar in both groups (approximately 7%). These findings are consistent with those of extensive databases at comparable follow-up periods, which did not reveal any significant discrepancies based on the modality of the administered technique. Data from the PARTNER 1 and 2 trials, which included patients with higher-risk profiles, failed to demonstrate any significant differences in the incidence of perivalvular leakage (PVL) [107]. Results from a cohort of patients with low-risk profiles who underwent treatment for aortic stenosis at two years postprocedure provided further corroboration for the hypothesis that there are no differences in the development of infective endocarditis (<1%) [108].

It seems pertinent to suggest that there may be certain factors that could be perceived as indicative of an elevated probability of PVE in each instance of therapeutic intervention and in each respective approach. In the context of TAVR, the following variables have been demonstrated to be associated with an elevated risk: lower patient baseline age at the time of the procedure, the necessity for postoperative pacemaker placement, and persistent periprosthetic valvular regurgitation.

In the context of surgically implanted valvular prostheses, the precise risk indicators linked to PVE have been identified as the type of prosthesis utilized and the occurrence of postoperative infections, including those affecting the sternum, urinary tract, or catheter-induced infections [109,110].

PVE affecting the pulmonary valve presents a unique set of challenges, particularly for younger patients who often require multiple prosthetic valve interventions. It would seem that there is an increased risk of PVE, particularly in connection with the use of several specific prosthesis valve. It has been suggested that there may be an association between complications and the transcatheter implantation of prosthetic valves, such as the Melody (Medtronic, Minneapolis, Inc., Minneapolis, MN, USA) valve [111,112]. A correlation has been established between the utilisation of Contegra (Medtronic, Inc.) valves following a surgical procedure and an elevated risk of PVE [113]. It is noteworthy that bovine jugular vein valves appear to exhibit a comparable propensity for PVE, irrespective of the method of implantation, whether surgical or percutaneous [114]. It is important to acknowledge that this type of prosthetic valve may carry a relatively high risk of prosthetic valve endocarditis, particularly in cases where the prosthetic graft has been in position for a duration of seven years or more [115].

## 4. Influence of the 2023 Duke/ISCVID IE Diagnostic Criteria on the Diagnosis of PVE

PVE should be diagnosed and managed by multidisciplinary endocarditis teams [8]. The 2015 European Guidelines contain proposals for the structure and tasks of a team [11]. In short, experts in cardiology, infectious diseases, cardiac imaging, cardiac surgery, and neurology will be needed. In addition to these core disciplines, geriatricians or addiction specialists should be taken into consideration, depending on the local epidemiology of PVE in the individual institution.

The 2023 Duke/ISCIID guidelines for the diagnostic evaluation of infective endocarditis represent a significant advancement in the diagnostic tools available for the clinical assessment of prosthetic valve endocarditis [7]. As primary indicators for diagnosis, they encompass intraoperative examination by an experienced surgeon, microbial characterisation through the utilisation of polymerase chain reaction (PCR), amplicon, or metagenomic sequencing in suitable samples, and the in situ hybridization of genetic material.

**Figure 4:**
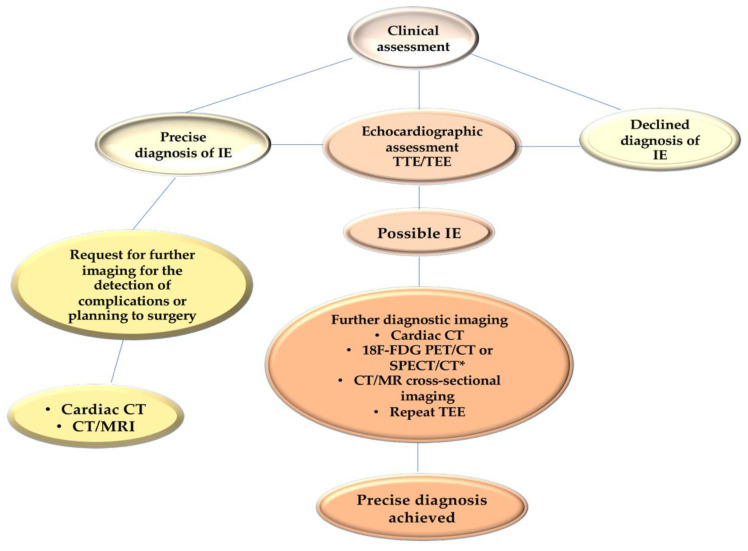
This figure shows the applied strategy of integrated imaging in patients with suspected infective endocarditis IE. In patients included in the subgroup with possible IE after initial evaluation by TTE and TEE, cardiac CT imaging, metabolic imaging, or transverse imaging of the head and viscera by CT scan or MRI is indicated to achieve a precise early diagnosis. For suspected IE 18 FDG-PET/CT or cross-sectional imaging by CT or MRI (or metabolic imaging), scans may assist with the detection of complications, such as abscess, mycotic aneurysm, infarct, or haemorrhage in patients with definite IE. Abbreviations: IE, infective endocarditis; FDG-PET/CT, fluorodeoxyglucose positron emission tomography/computed tomography; MRI, magnetic resonance imaging; TEE, transoesophageal echocardiography; TTE, transthoracic echocardiography. From Nappi et al. [7,36]. * Performed after TEE and discussed in Heart Team.

Furthermore, the list of bacteria includes those that are generally considered to be specific to the context of cardiovascular implants, such as coagulase-negative staphylococci and *Corynebacterium striatum*. Furthermore, the list includes *Corynebacterium jeikeium*, *Serratia marcescens*, *Pseudomonas aeruginosa*, *Cutibacterium acnes*, non-tuberculous mycobacteria (especially *M. chimaera*), and *Candida* spp. Furthermore, the findings obtained from CTA and 18F-FDG-PET/CT are included as principal criteria, with the same weight as that assigned to echocardiography [7,8] (Figure 4).

It is, therefore, recommended that TEE is performed in the majority of cases, even in instances where TTE has provided sufficient indications for a definitively conclusive diagnosis. The sensitivity of TTE in cases of suspected endocarditis in patients with prosthetic valves is poor, reaching a minimum of 36–69%. In such cases, proceeding with a TEE is usually the most suitable course of action [8,116,117,118,119]. In the presence of a heart device infection, the choice of TEE is also a priority. Furthermore, if a complication is suspected, TTE should be repeated, and at the completion of therapy, a baseline scan should be conducted for follow-up purposes [7,13,116,117,118,119]. Table 1 delineates the sensitivity and specificity of an echocardiographic diagnosis, with the objective of assessing the presence of an abscess.

Fluorine-18 fluorodeoxyglucose (18F-FDG) positron emission tomography (PET)/computed tomography (CT) has been incorporated into the 2023 Duke/International Society for Cardiovascular Infectious Diseases (ISCVIID) criteria as both a major and minor diagnostic tool. This is due to its efficacy in reclassifying cases from a “possible” to an “established” infective endocarditis (IE) diagnosis [7]. While it can prove challenging to distinguish between postoperative inflammatory uptake patterns, which are often diffuse and homogeneous, and infection, which is frequently intense, focal or multifocal, and heterogeneous, advances in digital positron emission tomography (PET) in conjunction with electrocardiogram-gated computed tomography angiography (CTA) are gradually addressing the shortcomings associated with early infective endocarditis (IE) within the initial three months of valve implantation [120]. In addition to its use in cardiac imaging, 18F-FDG-PET/CT is a valuable modality for detecting extracardiac findings associated with septic embolism, as well as for providing an alternative diagnosis for patients who have been classified as having “rejected” infective endocarditis (IE) [121]. Additionally, recent research indicates that 18F-FDG-PET/CT can serve as a reliable diagnostic tool for TAVR IE [122] and for monitoring the efficacy of antimicrobial therapy [123].

**Figure 5:**
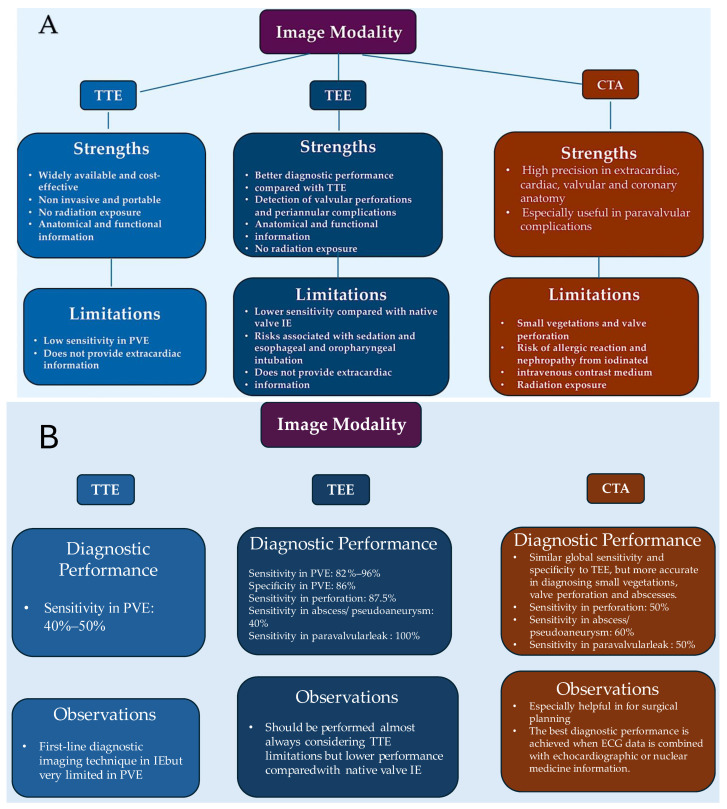

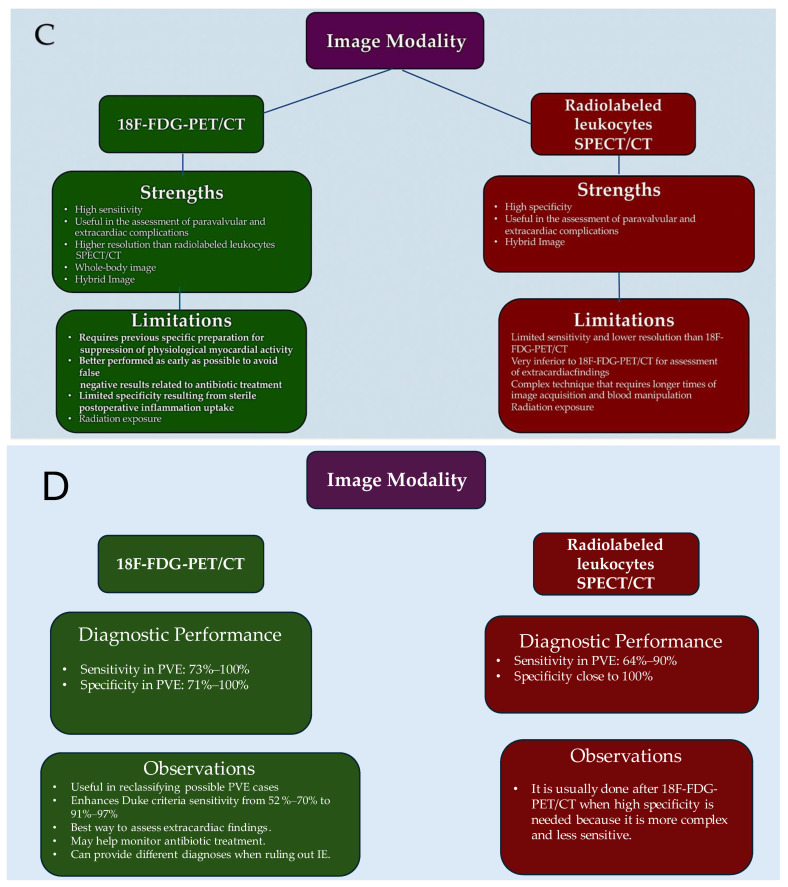
This illustration presents a comprehensive overview of multimodality imaging in the diagnosis of PVE (**A**–**D**), elucidating its characteristics, strengths, and limitations, as well as its diagnostic performance in TTE (**A**,**B**), TEE (**A**,**B**), CT (**A**,**B**), 18F-FDG-PET-CT (**C**,**D**), and radiolabelled leukocytes SPECT-CT (**C**,**D**). Abbreviations: ECG, electrocardiogram; IE, infective endocarditis; PVE, prosthetic valve endocarditis; SPECT-CT, single-photon emission computed tomography/computed tomography; 18F-FDG-PET-CT, fluorine-18fluorodeoxyglucose positron emission tomography/computed tomography [7,11,116,117,118,119,120,121,122,123,124].

Cardiac CTA is a valuable tool for diagnosing paravalvular complications. Additionally, it provides crucial extracardiac, cardiac, valvular, and coronary anatomical information, which is essential for surgical planning. To enhance the diagnostic performance of this hybrid imaging modality, it is recommended to combine it with nuclear medicine imaging techniques [117,118]. While radiolabelled leukocyte single-photon emission CT (SPECT)/CT has high specificity, it has been superseded by 18F-FDG-PET/CT as the preferred imaging modality due to its relatively higher technical complexity, lower sensitivity, and resolution [124]. Figure 5A–D illustrates the efficacy of a multimodal imaging methodology in diagnosing individuals with presumed PVE in alignment with international guidelines. The figures present a summary of the approach’s key strengths, limitations, diagnostic performance, and observations from the most recent conducted validation investigations into the diagnosis of prosthetic valve endocarditis [7,11,116,117,118,119,120,121,122,123,124].

**Figure 6:**
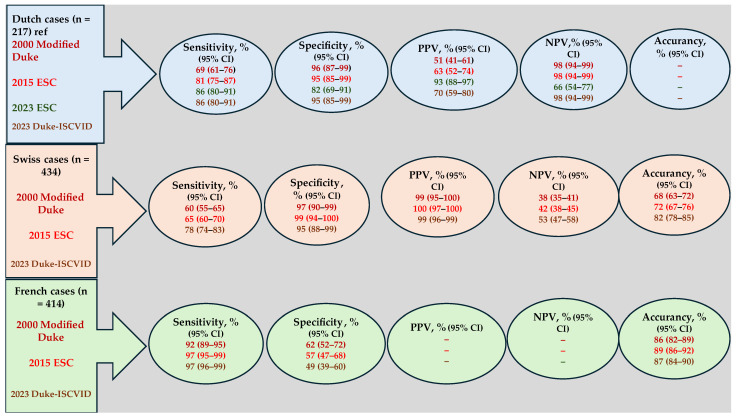
This illustration demonstrates the performance of the 2023 ESC and Duke/ISCVID diagnostic criteria among patients with prosthetic valve endocarditis and compares it with that of previous criteria. Abbreviations: ESC, European Society of Cardiology [7,8,11,125,126,127].

As a consequence, the 2023 Duke/ISCVID criteria for PVE exhibit enhanced sensitivity compared with the previous iteration and the 2015 European Society of Cardiology (ESC) guidelines, while retaining the same level of specificity [7,11]. The latest iteration of the 2023 ESC guidelines [8] incorporates numerous innovative features derived from the 2023 Duke/ISCVID IE criteria [7]. While the sensitivity for identifying prosthetic valve endocarditis is comparable, the specificity is somewhat diminished (Figure 6). Neither of the 2023 sets of IE diagnostic criteria has been employed for the evaluation of performance in the diagnostic evaluation of IE following TAVR. Table 2 provides a summary of the differences between these guidelines in terms of diagnostic criteria [7,8,11].

## 5. Treatment

### 5.1. Medication Control

In the absence of fungal PVE, there is no justification for a mandatory procedure on the grounds of a surgical approach in cases of PVE alone, unless there are other clear indications. The most significant published studies of recent years, which employ robust methodological designs (with appropriate adjustments for selection and immortalisation biases) [128,129], have reached the conclusion that surgical indications should be made on an individualised basis. This is based on the observation that a subgroup of patients may have favourable clinical outcomes with exclusive medical treatment. The positive effects of surgical intervention are more pronounced in patients who are more likely to undergo surgery (i.e., who have a greater need for it) than in those with less of a predisposition to surgery. It follows that any individual lacking a clearly defined surgical criterion (extending beyond the etiological factor) should first be considered for medical management as a primary option.

The 2023 ESC IE guidelines [8] represent a significant departure from previous recommendations for antimicrobial therapy in the initial crucial stage (the earliest 2 weeks of administration) of PVE [8,130]. However, despite this notable change in clinical practice, there are no major alterations to the existing recommendations for antimicrobial therapy in the initial crucial stage of PVE [9]. Patients with noncomplicated PVE who have commenced parenteral antibiotic therapy at the medical facility may conclude this phase of treatment in a domestic setting.

**Figure 7:**
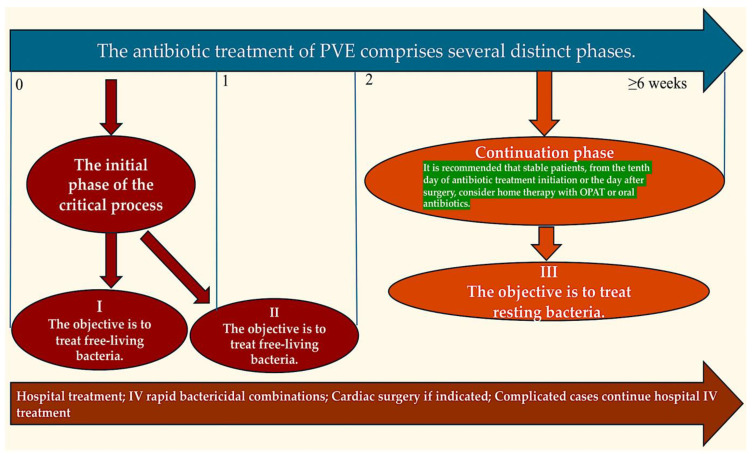
The accompanying illustration depicts the various phases of antibiotic treatment for prosthetic valve infective endocarditis. At present, two phases are under consideration for the treatment of prosthetic valve endocarditis: the first induction phase, which lasts for two weeks and is mandatory parenteral, and a second consolidation phase, in which some patients may be eligible for completion therapy through an outpatient parenteral antibiotic treatment (OPAT) regimen or orally. In this context, the term “free-living bacteria” refers to bacteria in the active replication phase, while “resting bacteria” are bacteria in the quiescent or dormant phase.

New guidelines have been established for the treatment of PVE at home [9]. These guidelines differentiate between oral therapy and outpatient parenteral antibiotic therapy (new GAMES criteria). Following the commencement of intravenous antibiotic therapy at the hospital, a subset of patients with noncomplicated PVE may complete their antibiotic treatment at home, either with outpatient parenteral antibiotic therapy (OPAT) or orally (Figure 7).

In a study conducted by Pericas et al. [9], data from the Spanish GAMES registry were analysed. The study included 429 patients with IE who had completed antibiotic treatment under an OPAT regimen. Patients with a history of injection drug use were excluded from the study. Despite PVE being an exclusion criterion for outpatient management in accordance with American and European guidelines, 117 patients in the study had PVE. It is noteworthy that the study did not demonstrate an enhanced mortality rate or an increased incidence of hospital readmission. In light of these findings, the investigative team put forth a novel proposal for the identification of patients with PVE who would benefit from OPAT treatment. This proposal, termed “OPAT-GAMES”, represents an extension to the existing criteria for patient selection. In a recently conducted evaluation study utilising the GAMES registry, Pericas and colleagues [9] were able to substantiate the efficacy of the aforementioned criteria. This was evidenced by the observation that patients undergoing OPAT for IE exhibited a comparable incidence of complications and hospital readmissions to that observed in the general population [9]. Furthermore, dalbavancin, a long-acting glycopeptide, has the potential to be employed in the OPAT consolidation treatment [37] (Table 3).

The POET trial delineated oral antibiotic regimens utilized as transitory therapy in the treatment of infective endocarditis (IE). The trial encompassed the assessment of 2000 patients over a five-year period. A total of 400 patients were selected for the study, 107 of whom exhibited the presence of PVE. These patients were randomly assigned to either an oral or parenteral treatment group, with 54 individuals receiving oral treatment and 53 receiving parenteral treatment [10]. The trial did not yield statistically significant findings regarding the primary endpoint (composite of all-cause mortality, unplanned cardiac surgery, embolic events, or relapse of bacteraemia with the primary pathogen) from the randomization period to six months post antibiotic treatment completion. However, it is important to note that the trial was not designed with sufficient statistical power to assess the specific subgroup of patients with PVE.

In summary, the treatment of PVE may be conducted either intravenously with outpatient parenteral antibiotic treatment (OPAT) or orally. In accordance with the recently established OPAT-GAMES (Grupo de Apoyo al Manejo de la Endocarditis Infecciosa en España) criteria [9] and the POET (Partial Oral Treatment of Endocarditis) trial, the present study employs a methodology consistent with the aforementioned criteria [10].

### 5.2. Surgery and the Role of Catheter-Based Treatment Therapy for PVE on Bioprosthetic Valves

In patients with PVE, surgical intervention during the primary is indicated in cases where prosthetic valve dysfunction has resulted in heart failure. There is a paucity of significant amendments to the surgical management paradigm [131]. Owing to the constraints imposed by the stipulated word limit, a detailed account of the diverse operative alternatives, the function of heterografts, and the potential for heart transplantation in highly circumscribed instances is omitted. Nevertheless, the elevated prevalence of comorbidities and the concomitant surgical hazard may preclude surgery in some patients, particularly those with transcatheter valves, despite indications for surgical option [30,132]. The use of transcatheter interventions has been identified as a possible alternative to surgical treatment for patients who have been deemed to have a high surgical threshold and who are suffering from severe residual valvular dysfunction. These include techniques such as percutaneous repair of paravalvular leak, transcatheter edge-to-edge mitral valve repair, and aortic and mitral valve-in-valve replacement, which may offer an alternative treatment pathway to surgery. The majority of these procedures were conducted in patients who were not infected at the time of the procedure [133,134]. In instances where it is feasible to do so, antibiotic medication may be deferred until such a time as there are no remaining indications of active infection, and the course of antibiotic administration has been fulfilled. There is a paucity of empirical evidence to support the efficacy of antimicrobial therapy during the active phase of endocarditis, with only a limited number of documented cases having been reported in the literature [135]. In order to ascertain the potential benefits and risks of prolonged oral antimicrobial therapy in specific cases, it is recommended that collaboration with an infectious diseases specialist be sought, with due consideration given to the specific pathogen involved. Table 4 presents a comparative analysis of the various surgical criteria employed in the diagnosis of infective endocarditis. The use of colour-coding allows for the differentiation between the three distinct classifications.

### 5.3. PVE in the TAVR Era: New Challenges

For persons suffering from aortic valve stenosis who were previously deemed unsuitable for surgery, TAVR has significantly improved the prognosis [136,137]. It should be noted that the patient profile for TAVR is often characterised by frailty, multiple medical interventions, and an elevated risk of bacteraemia and IE. However, the technology is expected to become more widely available over time, extending to intermediate and low-risk populations. The management of TAVR endocarditis presents a significant challenge to cardiologists and surgeons in the contemporary management of IE. This is particularly the case in elderly patients with high-risk profiles who are unsuitable for surgical intervention and for whom the optimal approach to managing PVE remains unclear. What is the role of surgical intervention in this context?

A limited number of patients with infective endocarditis following TAVR were disclosed in the seminal Placement of Aortic Transcatheter Valve (PARTNER) trials [136,137]. The occurrence and outcomes of TAVR endocarditis are now being elucidated by observational data cohorts drawn from the real world. In a single-centre study conducted in Germany, 55 cases of TAVR endocarditis were reported, representing a cumulative incidence of 3.02%. When expressed per patient-year, this incidence was found to be 1.82% [138]. Of the total number of cases, 42% (23 of 55) were identified as healthcare-acquired. The results of the multivariate analysis indicated that chronic haemodialysis and peripheral arterial disease constituted significant risk factors for the subsequent occurrence of TAVR endocarditis. Chronic haemodialysis exhibited a hazard ratio (HR) of 8.37 and a 95% confidence interval (CI) of 2.54 to 27.63, while peripheral arterial disease displayed a HR of 3.77 and a 95% CI of 1.88 to 7.58. A total of 38% of cases were caused by *Staphylococcus aureus*, 31% by enterococci, 9% by coagulase-negative Staphylococci (CoNS), and 9.1% by Streptococci. In seven cases, an infectious process affecting the prosthetic valve was superimposed on a primary valve infection [138].

A supplementary report [139] detailed the cases of 53 patients who had been diagnosed with TAVR endocarditis within a multicentre U.S. registry. This represented a cumulative incidence of 0.67% at a mean follow-up period of 1.1 years. In the initial postprocedure period spanning the first year, a prevalence of 0.5% for TAVR endocarditis was observed, with a median time to occurrence of 6 months. A majority of patients (70% or greater) presented with a fever, while 77% exhibited a discernible vegetation on echocardiographic imaging. The investigation revealed that approximately 50% of patients exhibited bacteraemia, with the probable cause identified as an antecedent procedure. Furthermore, the findings indicated that the administration of antibiotic prophylaxis was employed in 59% of cases. The most frequently identified causative pathogens were CoNS (25%), *Staphylococcus aureus* (21%), and *Enterococcus* (21%). Although the self-expanding CoreValve system (Medtronic, Minneapolis, Minnesota) emerged as an independent risk factor for IE (hazard ratio [HR]: 3.1; 95% confidence interval [CI]: 1.37 to 7.14), further validation in other series is required before this finding can be accepted [139].

Raguiero and colleagues [30] conducted a detailed analysis of the data compiled by the Infective Endocarditis after TAVR International Registry. This registry included reports derived across 47 centres situated in various global locations, with a total of 250 cases being encompassed by the registry. The global incidence across the entire cohort amounted to 1.1% per person-year, with a median time to presentation of 5.3 months following the procedure. A multivariate analysis identified the subsequent factors as predictive: younger age (hazard ratio, HR: 0.97 per year; 95% confidence interval, CI: 0.94 to 0.99), male sex (HR: 1.69; 95% CI: 1.13 to 2.52), diabetes mellitus (HR: 1.52; 95% CI: 1.02 to 2.29), and moderate-to-severe aortic regurgitation (HR: 2.05; 95% CI: 1.28 to 3.28). The primary infectious agents identified were enterococci (24.6%) and *S. aureus* (23.3%). The observed case fatality rate was 36% at the time of hospital admission, rising to 67% at two years. It seems likely that further patient- and device-related factors that contribute to an increased risk of endocarditis will also be identified. This may also facilitate a greater understanding of the nature of endocarditis. The elevated incidence may also be attributed to an elevated probability of adverse outcomes during the initial postoperative months. To enable a comparison with the outcomes of surgical valve replacement, a longer follow-up period is necessary [30].

More recently, del Val and colleagues [140] analysed data from the Infectious Endocarditis after TAVR International Registry. Their study cohort comprised patients with confirmed IE following TAVR from 59 centres in 11 countries. Patients were classified into two groups based on the microbiological aetiology: non-*S. aureus* infective endocarditis and *S. aureus* infective endocarditis. A total of 141 patients out of 573 (24.6% of all cases) were found to have had a staphylococcal IE. In the majority of cases (115/141, 81.6%), the infecting strain was methicillin-sensitive *Staphylococcus aureus*. Self-expanding valves were employed with greater frequency than balloon-expandable valves among patients with an earlier diagnosis of IE caused by *S. aureus*. The occurrence of severe bleeding and sepsis in conjunction with TAVR, as well as the onset of neurological symptoms or systemic embolism at the time of admission, and the presence of IE with cardiac device involvement (excluding the TAVR prosthesis) were identified as risk factors for *S. aureus* IE (*p* < 0.05 for all). In the group of patients with IE subsequent to TAVR, the probability of *S. aureus* IE increased from 19% in the absence of the aforementioned risk factors to 84.6% when three risk factors were identified. Patients with *S. aureus* IE exhibited elevated rates of inpatient mortality (47.8% vs. 26.9%; *p* < 0.001) and 2-year mortality (71.5% vs. 49.6%; *p* < 0.001) compared with those with non-*S. aureus* IE. The administration of surgical intervention during the initial episode of *S. aureus* IE was associated with a reduction in mortality at the follow-up stage in comparison with the sole use of antimicrobial medication (adjusted hazard ratio 0.46, 95% CI 0.22–0.96; *p* = 0.038) [140]. Table 5 presents a selection of the most pertinent studies on TAVR-IE.

## 6. Short and Long-Term Results

**Figure 8:**
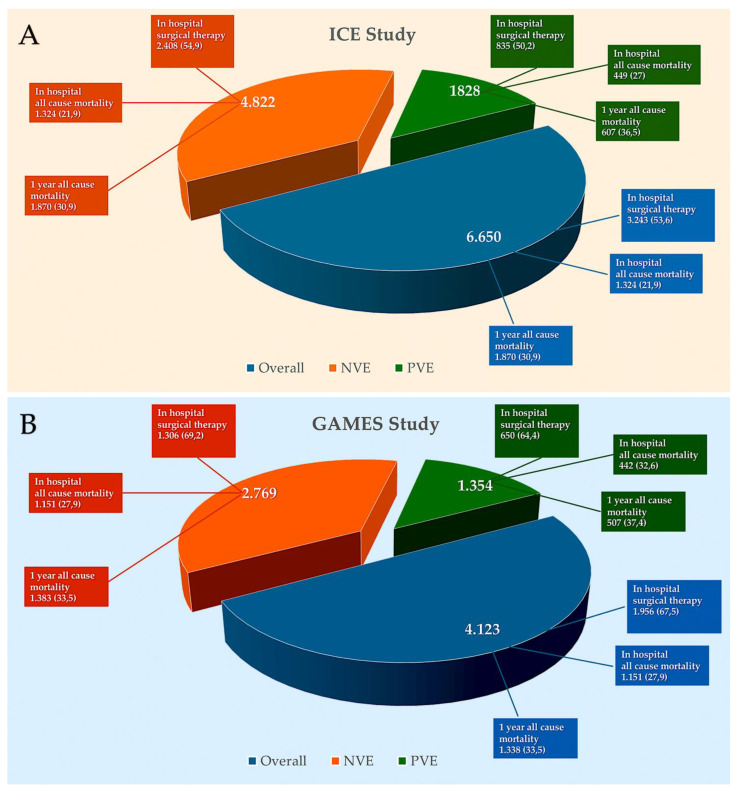

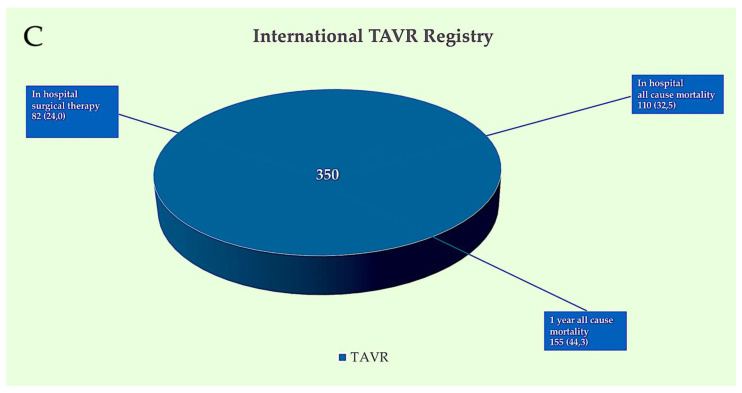
(**A**–**C**) This illustration presents the overall clinical outcomes reported in multicentre cohorts ((**A**), ICE study; (**B**) GAMES study) with a comparison of native, prosthetic, and TAVR international registry endocarditis (**C**). The values are expressed as a percentage (n). The N value refers to patients with infective endocarditis involving the aortic prosthesis [29,30,31].

Figure 8A–C illustrates the incidence of in-hospital surgery and mortality rates, with 1-year mortality data for PVE from several previously published studies [29,135,147].

The prognosis for patients with PVE with definitive indications is poor in the absence of surgical intervention [148]. Regrettably, perioperative mortality remains exceedingly considerable (>25%) in the majority of cases [149]. This has prompted a revaluation of the necessity of surgical intervention during the acute phase of infection in these instances [125,126]. Nevertheless, in a comprehensive retrospective analysis, 73% of patients with PVE who proceeded to surgery exhibited a notable survival advantage (mortality 9% vs. 34%; *p* < 0.001). The results demonstrate that prosthetic aortic valve implantation in patients with PVE treated with cryopreserved human allograft implant has an excellent in-hospital survival rate, reaching 96.1%, and a 10-year survival rate of 56% [135]. It is, however, important to note that positive outcomes may be less evident in centres with less experience in performing the procedure.

### 6.1. The Choice of Valve Substitute

The utilisation of human cryopreserved homograft in the initial aortic valve replacement for IE demonstrated a decline over time, with a reduction from 9.4% to 5.6%. Similarly, there was a decrease in the use of homograft in reoperation, from 37.5% to 28.5%. The findings presented here are based on a report from the STS database covering the period between 2005 and 2011. Nevertheless, the utilisation of a homograft was more common in reoperations than in primary interventions (32.2% vs. 7.0%, *p* < 0.0001). This encompassed both valve replacements (14.6%) and root replacements (53.2%) [150]. The potential for the use of a homograft in IE to improve clinical outcomes continues to be inconclusive due to the absence of randomised controlled trials (RCTs) [19,151,152,153,154].

No significant discrepancies have been identified in the observed mortality and reinfection rates in patients with infective endocarditis who have undergone replacement of the affected valve with either a mechanical or a biological substitute [22,151,152,155,156]. In a similar report by Klieverik et al. [155], the recurrence of endocarditis in recipients of homografts was found to be comparable with that of mechanical valves, with a reduced rate of patients remaining free from reoperation (76% vs. 93%, respectively). As reported by Sabik and colleagues, [153] a total of 103 patients with prosthetic infective endocarditis underwent aortic homograft surgery. Of these patients, 78% had periannular and root abscesses. The study demonstrated that more than 95% of patients remained infection-free for over two years postoperatively, with only 3.9% of cases resulting in mortality during surgery [153].

The Fukushima et al. [157] study demonstrated a low reinfection rate (0.2%) at 30 days and a late infection rate of 5.5% with a median time of 5 years (4 months to 16 years) post allograft implantation. In a study by Arabkhani et al. [158], aortic homografts yielded favourable results 27 years postoperatively, with only a 2.2% recurrence rate of reoperation due to relapsing infections. Antibiotic treatment has been demonstrated to be an effective approach in 21–25% of cases involving allogenic tissues [158,159]. Musci et al. [159] conducted a study encompassing 1163 cases and reported the outcomes of 221 aortic root replacement surgeries utilising aortic homografts. The findings revealed that patients with active endocarditis and periannular abscess formation exhibited a significantly reduced rate of reinfection (5.4%), both in cases of native valve endocarditis and in those involving prosthetic valves. Additionally, the 10-year reoperation-free survival rate was 92.9% ± 3.2% and 92.1% ± 2.5%, respectively. The mortality rate at an early stage was 16% for NVE and 25% for PVE. There was, however, an improvement in 10-year survival rates, with 47% for NVE and 35% for PVE. A significant proportion (25%) of deaths were recorded during the course of the operation, which serves to illustrate the inherent complexity of this surgical procedure in patients who are critically ill [159]. The clinical performance and durability of the treatment demonstrated by Yanka et al. [160] was excellent, with a low rate of reinfection and a late mortality rate of 7.9%. At the one-year mark, patient mortality was recorded at 97%, with the figure dropping to 91% at the ten-year mark. In a study by Perrotta et al. [161], the five-year cumulative survival rate for homografts was 88%, compared with 66% for prostheses in PVE. In their research, Kim et al. [22] demonstrated that the risk of reinfection within one year was lower in patients who had undergone homograft surgery.

The utilisation of a homograft as a substitute for aortic and mitral valve disease was reported in 56.2% and 21% of patients with abscess formation [14,19,162]. In cases where an aggressive IE was present with involvement of the intervalvular fibrosa and mitral valve, a double homograft was employed as a surgical intervention [5,6,7,8,9,10,11,12,13,14,15,16,17,18,19,20,21,22,23,24,25,26,27,28,29,30]. Monobloc implants were performed in two-thirds of cases, while the remaining patients received a separate bloc with partial mitral homograft insertion [14,19,162]. The results of the implant technique have been favourable even in cases where there was fragile tissue present [163,164].

The employment of an allogeneic substitute in the context of broad base infections of the cardiac structure, irrespective of whether the affected valve is native or prosthetic, is supported by the findings of Steffen et al. [165]. Cryopreserved aortic homograft (CAH) demonstrates antibacterial efficacy, despite a conservation period of up to five years. The antibiotic combinations applied during the processing of cryopreserved aortic homograft have a significant effect on the resistance of these grafts to infection. The tissue from the ascending human allograft has been observed to demonstrate significantly elevated bacterial resistance against staphylococcal bacteria, including *S. epidermidis* and *S. aureus*, when compared with those of homograft aortic valves. This has been accompanied by a concomitant reduction in bacterial contamination. The application of antibiotics subsequent to the thawing of CAH has resulted in a notable reduction in the recurrence of infection, a feat that conventional prostheses and Dacron grafts have yet to achieve. Nevertheless, the risk of vascular graft infection can be mitigated by the prior administration of antibiotics [165].

In the period preceding the year 2000, the use of mechanical prostheses was observed in 50% of patients, compared with a significantly lower rate of 14% since 2009. A review of the STS Database [150] revealed a notable shift towards biological valves during the period between 2005 and 2011. This trend was observed in both primary (NVE) and reoperation (PVE) procedures. In the primary operation, biological valves were used in the majority of cases, representing 8421 patients (approximately 73%) compared with mechanical prosthesis. In the reoperation setting, biological valves were utilized in 3139 patients (approximately 27%) compared with mechanical prosthesis. The use of biological prosthetic devices rose from 57% to 67% for the primary operation, while the use of mechanical prosthetic devices declined from 30% to 24%. With regard to repeat operations, there was an increase in the utilisation of biological prosthetic valves, from 38% to 52%, in comparison with a decline in the utilisation of mechanical prosthetic valves, from 20% to 17%. Only 2.5% of all valve replacements involved the use of a homograft. Conversely, biological valves were employed in 68.7% of cases. The aforementioned trend was contradicted in both NVE and PVE cases where the aortic root constituted the site of involvement [150,153].

The findings of Moon et al. [152] indicated an ambiguous risk of repeat surgery for infection recurrence among patients with mechanical (2.1%) and bioprosthetic valves (2.3%) at five years. Beyond this timeframe, a marginally elevated risk was observed for mechanical prostheses (0.5%) in comparison with stented xenografts (1.1%). A comparable prolonged postoperative survival rate, excluding operative mortality, was observed between mechanical prostheses (62% and 46%) and bioprosthetic prostheses (61% and 41%) at 10 and 20 years, respectively [152]. In cases of endocarditis complicated by abscess formation, Kim et al. [22] opted for the use of mechanical prostheses over stented xenografts. Furthermore, this trend was observed in cases where there was concomitant involvement of the intervalvular fibrosa and the mitral valve (mechanical valves in 38% of patients compared with xenografts in 18.7%) [22]. These findings are in alignment with those previously documented by David et al. [151], which outlined favourable outcomes when mechanical valves were employed in conjunction with synthetic patch or prosthetic valve conduits within the context of difficult-to-manage aortic valve endocarditis [151].

**Figure 9:**
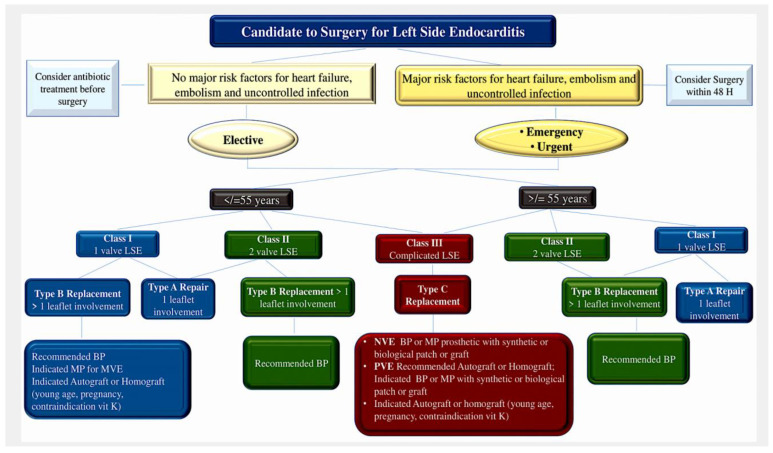
The following illustration presents the key take-home messages and clinical algorithm for the management of left-side endocarditis, based on the 2023 Duke/International Society for Cardiovascular Infectious Disease (ISCVID) criteria as outlined by the ESC in 2023. Abbreviations: BP; bioprosthesis; GMT, guide medical therapy; MP; mechanical prosthesis; MVE, mitral valve endocarditis; NVE, native valve endocarditis; PVE, prosthetic valve endocarditis.

**Figure 10:**
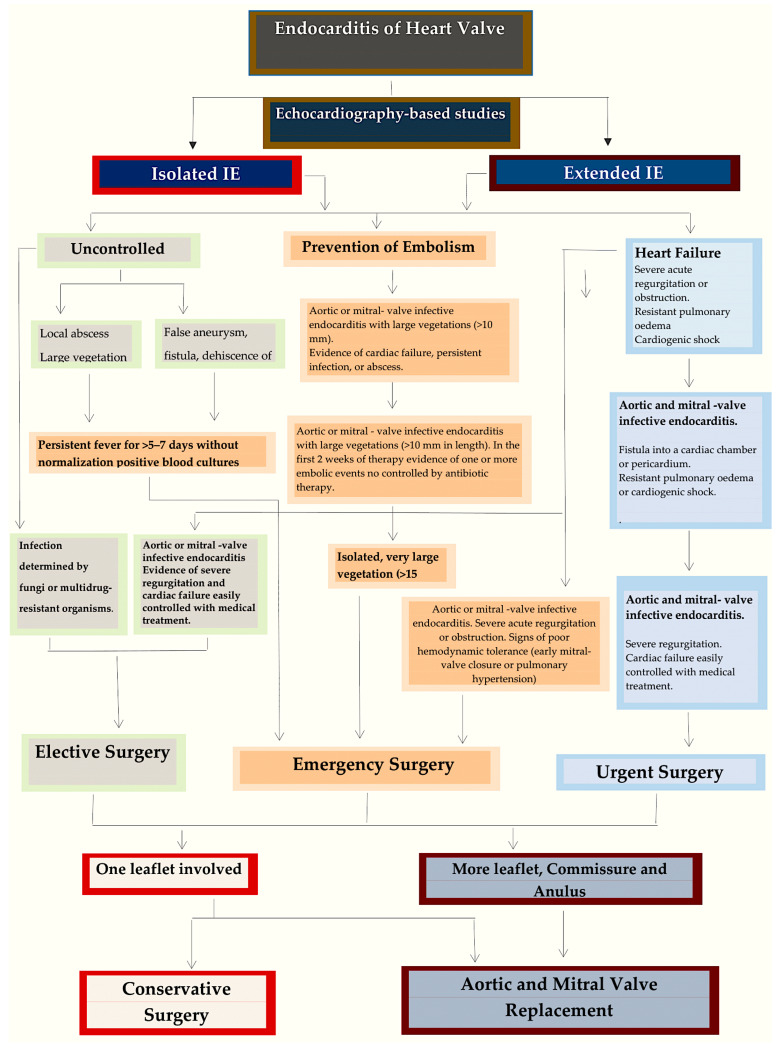
A methodology for the treatment of heart valve endocarditis. TEE ultrasound enables the identification of three categories of patients for whom surgical intervention is recommended and involving elective (grey box), urgent (rose box), and urgent (blue box). The extent of the infectious process is the criterion that guides the choice between surgical options, with the aim of tailoring the conservative or extensive approach towards repair or replacement. The surgical option to be pursued, whether repair or replacement, is determined by the clinical and anatomic findings of the preoperative imaging. In the event of an infection process being confined to a localised area of the valve leaflet, it would be prudent to consider conservative surgery with aortic valve repair (illustrated by the light rose box). In cases of extensive infectious damage with severe pathoanatomical compromise of the valve, surgical replacement of the aortic valve is the recommended course of action (dark grey box). It is imperative that a multidisciplinary team makes a joint decision regarding the timing. In the case of an emergency surgical option (illustrated in the pink box), the surgical procedure to remove the infected valve must be requested within 24 h of the completion of the diagnostic procedure. In the case of the urgent surgical option (blue box), the procedure should be carried out within a few days of the indication being given. In patients who require the elective surgical option (green box), the procedure should be performed after a minimum of one to two weeks of antibiotic administration [22,97,153,154].

Operative options in patients with aortic PVE vary, depending on the depth of involvement at the aortic annulus, extension towards the intervalvular fibrosa body and other regions of the fibrous skeleton of the heart. Extensive aortic root involvement is managed by exteriorizing necrotic areas to the pericardium as part of the reconstruction. The potential benefits of this strategy include a negligible risk of recurrence and a decreased risk of periprosthetic leak or late pseudoaneurysm formation. Moreover, a low threshold for full aortic root replacement translates into better debridement, superior anchorage, and improved haemodynamic performance (through allografts or the ability to implant larger prosthetic valves) [154]. The advantages of homograft root replacement over other techniques include better pliability and adaptation to damaged aortic annuli/left ventricular outflow tract tissue, which may translate into superior haemostasis. Additionally, superior valve haemodynamics and lower thrombogenicity can improve early postoperative outcomes. In-hospital survival for prosthetic aortic valve replacement in cases of PVE treated at the Cleveland Clinic with human allograft replacement was encouraging (96.1%) [153]. Isolated prosthetic mitral valve endocarditis is a different situation [156], and most patients can be offered straightforward repeat mitral valve replacement. There has been significant debate on whether different replacement devices have been associated with relapses. To date, there is no evidence of superiority for any of the available options (mechanical, bioprostheses, and allografts) [22,97] (Figure 9 and Figure 10).

### 6.2. Infective Endocarditis in TAVR Patients

A study conducted in France utilised data from a national database (EuroSCORE II mean, 3.5%) to demonstrate that the occurrence of PVE following TAVR is associated with an elevated mortality rate when compared with SAVR (43% vs. 32%; relative risk [RR] = 1.32; 95% CI: 1.08–1.60) [166]. Moreover, in one of the most extensive databases pertaining to TAVR procedures, the incidence of periprosthetic valve infection necessitating valve explantation was observed to be merely 14.8% in a patient cohort. Notably, there was no statistically significant correlation between surgical intervention and the reduction in mortality (29.7% for surgery vs. 37.1% for nonoperative management; *p* = 0.39). A similar analysis indicates that at least 50% of patients with IE following TAVR require surgical intervention. However, only 16.4% of these patients ultimately undergo surgery [167]. A subsequent contemporary nationwide experience with TAVR IE yielded a cardiac surgical intervention in only 3.7% of TAVR PVE patients. The heightened prevalence of comorbidities and increased age of patients with IE subsequent to TAVR, as documented thus far, are presumed to underpin the low incidence of cardiac surgical procedures and the elevated in-hospital mortality rates observed in this cohort relative to other IE groups [168].

Several studies published recently found no evidence that the surgical management of PVE in recipients of a TAVR offers a survival advantage over medical management alone [110,132,169,170].

A recent multicentre German study investigating surgical experience in patients with IE following TAVR has revealed a median EuroSCORE II and STS PROM (Society of Thoracic Surgeons Predicted Risk of Mortality) of 17% and 3.1%, respectively. The respective survival rates at discharge and one-year postprocedure were 88.4% and 53% [171]. Although these figures are encouraging, they nevertheless give cause for concern when one considers the expertise of the participating institutes and the potential for biases in the selection of treatments [171].

## 7. A Multidisciplinary Team Approach to Decision Making in the Area of PVE

It is of the utmost importance to perform surgery at the optimal time for patients with native valve endocarditis or PVE. Postponing the procedure often results in a significant increase in the probability of adverse outcomes and an increased likelihood of mortality and morbidity during the procedure. Prior to the establishment of the heart team, which is responsible for coordinating patient care, it was common practice for surgeons to be consulted only when patients had failed to respond to medical therapy, were in a state of intractable heart failure, or had experienced extensive stroke or multisystem organ failure. This was despite the fact that cardiologists and other hospitals were already referring patients with IE to surgeons at an earlier stage. There is often a paucity of detailed knowledge surrounding the surgical challenges, the subsequent complications, and the clinical trajectory of these cases following surgical treatment. When coupled with the inherent difficulty in identifying the etiological agent, this frequently results in the postponement of surgical consultation, with patients presenting for surgery at a late stage and exhibiting markedly compromised clinical conditions, thereby increasing the risk of intraoperative complications [172].

It is, therefore, imperative that an experienced cardiac surgeon be consulted at the earliest possible stage to ascertain the optimum surgical option and timing, in order to ensure the best possible outcome for patients with prosthetic mitral valve endocarditis. For instance, it has been established that the risk of stroke is significantly elevated within the initial two-week period of antibiotic medication, particularly in cases where the infection is located on the left side of the heart and specifically in instances where it affects the mitral valve [14,173]. Furthermore, the potential for valve repair in lieu of replacement can only be considered following a detailed discussion between experienced surgeons and echocardiologists. Given that valve repair may result in superior long-term survival and functional outcomes compared with valve replacement, a collaborative approach involving a heart team is essential for the effective management of PVE [14,174].

In this context, the application of the Society of Thoracic Surgeons (STS) risk scoring system represents a valuable addition to the discussion with other specialists, as it facilitates the objective assessment of the surgical hazard and provides enhanced insight into potential perioperative complications [175]. Infectious disease experts should be integral members of the multidisciplinary team, offering essential guidance on the selection, dosage, and administration of antimicrobial agents, including antibiotics and antifungal drugs, to ensure optimal efficacy and minimize adverse effects. These healthcare professionals can also provide indispensable support in the treatment of antibiotic-resistant organisms or issues arising from the prolonged use of antibiotics. It is essential to include specialists in internal medicine, nephrology, obstetrics, and geriatric medicine as members of the heart team. In order to ensure the best possible decision-making process, it is essential that the input from all relevant disciplines is considered and that the individual characteristics of the patient are at the centre of the decision-making process. It is also important that the specialist dealing with the case addresses any specific aspects that may require attention [14,176].

**Figure 11:**
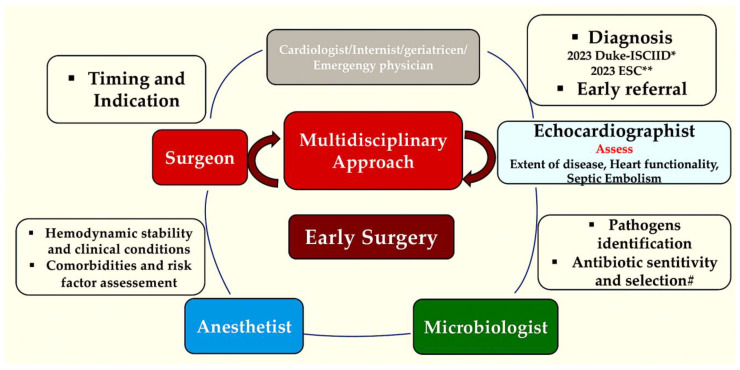
The illustrations present a clinical perspective and key messages derived from this review, emphasizing the most significant novel developments pertaining to the management of PVE. A coordinated, multidisciplinary approach to the diagnostic work-up and the necessity for prompt surgical referral, particularly in conditions with a high risk of embolisation and clinical deterioration with indications of heart failure, despite optimal antibiotic therapy, is advocated. PVE is a complex disease, and its management should, therefore, be undertaken on a multidisciplinary basis, with different professionals contributing their expertise to the decision-making process. It is proposed that a diagram should be constructed that highlights the role of each professional. It is recommended that the coordinated efforts of the team members converge towards the early referral of the patient to specialised centres with the aim of performing surgery as soon as it is feasible, according to the patient’s condition. It is advised that the surgical decision is not delayed, especially in cases of complex and extensive endocarditis, where radical and demolition surgical approaches with the use of homograft are advised [14,19,22,153,154,159,160,163,165]. The appropriate expert will be involved according to the individual characteristics, clinical needs, or comorbidities of each patient. * Ref [7]; ** Ref [8]; # Ref [9,10,37,125,126].

It is of the utmost importance to consider the specific needs of young women of potential reproductive age, particularly in the context of valve replacement. In such instances, the use of anticoagulant medication is not advisable, necessitating the delivery of bespoke counselling and discussion regarding the optimal strategy for valve replacement. Likewise, patients scheduled to undertake long-term dialysis should be assessed by a nephrologist prior to surgery, with a comprehensive plan for postoperative haemofiltration in place (Figure 11)

### When to Have Surgery?

Many authors have created models to help decide if IE is risky [175,177,178,179,180,181,182]. Gaca et al. made a score based on the Society of Thoracic Surgeons’ database [175]. It includes 13 risk factors for mortality, such as emergency status, cardiogenic shock, haemodialysis, and “active endocarditis”. Other studies have identified other factors affecting the type of valve infected and the causing bacteria [177,178]. The EuroSCORE (European System for Cardiac Operative Risk Evaluation) categorises “active endocarditis” as a patient who is still undergoing antibiotic treatment at the time of surgery. This indicates that the risk of undergoing surgery is high [177]. The PAL-SUSE score incorporates a number of predictive factors, including age of 70 years or above, extensive intracardiac destruction, infection with *Staphylococcus aureus*, the need for immediate surgical intervention, and sex assigned at birth as female [178].

The European Society of Cardiology [6,11] and the American College of Cardiology/American Heart Association (ACC/AHA) [5] have established a classification system for surgical indications based on the level of evidence. It is noteworthy that there is often a discrepancy between the guidelines and evidence-based data in the real world [178]. Liver disease was identified as the primary predictor of nonsurgical referral, with an odds ratio (OR) of 0.16 and a 95% confidence interval (CI) of 0.04 to 0.64. *Staphylococcus aureus* infection was also identified as a significant predictor, and the OR of the main predictors of nonsurgical referral were liver disease (odds ratio [OR] for surgery: 0.50; 95% CI: 0.30 to 0.85), stroke before surgical decision (OR: 0.54; 95% CI: 0.32 to 0.90), and other factors [178]. In contrast, individuals presenting with severe aortic regurgitation, abscess formation, or embolization were more likely to be referred for surgical intervention, as reported in the original study [178].

In patients who have experienced a cerebrovascular accident, surgical intervention should not be deferred in the absence of coma and intracranial haemorrhage (Class IIa, Level B). Conversely, in cases of minor cerebrovascular incidents, such as transient ischaemic attacks or silent cerebral embolism, surgical intervention should be promptly recommended (Class 1, Level B) [6,11]. Surgery should be delayed for at least 1 month in the case of devastating neurological events, such as intracranial haemorrhage and cerebral localisation of septic emboli with haemorrhagic evolution. CT scans or radionuclide perfusion scans should be conducted to evaluate lesion evolution according to the guidelines (Class IIa, Level B) [6,11]. Okita et al. demonstrated that early surgery (<7 days) is both a safe and effective procedure in Classes I and IIa, Level B patients who do not have a history of preoperative haemorrhagic stroke [183]. In addition to the development of a mycotic aneurysm, repeated CT scans immediately prior to surgery can exclude a preoperative haemorrhagic evolution of a cerebral infarct [184].

In accordance with the prevailing guidelines, the decision-making process concerning valve surgical strategies in IE should encompass a comprehensive evaluation of the long-term durability of the biological implants, the potential relapse of infection, and the risk of requiring additional surgery, which is often associated with detrimental effects on cardiac structure and function [5,6,11]. In light of the aforementioned considerations, it is essential to ascertain an appropriate balance between the potential risks and benefits when discussing surgical alternatives with the patient or their family. The principal risk is the deterioration of the prosthetic valve, which may result in valve failure and the necessity for further complex redo surgery and tissue demolition. Conversely, the benefits of this procedure are represented by a very low incidence of infection relapse [19,20,21,157,158,162,163,164]. However, when considering the short term, the primary issue in patients undergoing treatment for complex aortic or mitral PVE, which is frequently associated with mitro-aortic continuity and evidence of fistula formation into a cardiac chamber or pericardium, is not stroke-like episodes of structural valve deterioration (SVD) but rather the recurrence of infection [19,152,159,160,161,185,186]. A number of studies have demonstrated that extensive and radical surgery is often required for patients with IE who are receiving CAH. However, it has also been shown that mechanical prosthetic or stented bioprosthetic valves are used with comparable regularity in cases of complex endocarditis [19,22,163].

The prevalence of abscess formation in the patient cohort was observed to range from 40% to 67%, which is consistent with the severe nature of the disease being treated. The mortality rate associated with repeat surgical procedure for a recurring infection is higher than that observed in cases of reoperation for SVD or the dysfunction of an aortic homograft inserted in the aortic root position. In a significant number of patients with extensive valve endocarditis, some investigators have reported a greater propensity for the utilisation of mechanical prosthetics as a substitute for an infected aortic valve in comparison with the stented bioprosthetic (40.5% vs. 29.5%). This trend was also corroborated by the evidence of concomitant involvement of the mitral valve, with a prevalence of 38% compared with 18.7% [22].

The mortality rate associated with repeat surgery for a recurrence infection is elevated in comparison with the mortality rate associated with surgical reintervention for SVD or the dysfunction of a CAH positioned within the aortic root. In a significant number of patients with extensive IE, some investigative teams have indicated a preference for the use of mechanical prosthetic as a substitute for an infected aortic valve in comparison with the stented bioprosthetic valve (40.5% vs. 29.5%). This trend was also corroborated by the evidence of concomitant involvement of the mitral valve, with a prevalence of 38% compared with 18.7% [22,151]. While some studies have highlighted the favourable long-term outcomes of mechanical prosthetics, it is important to acknowledge the necessity for lifelong anticoagulation, which carries significant risks. Furthermore, the demographic most commonly affected by endocarditis is relatively young and is, therefore, inclined to pursue an active lifestyle. For patients, oral anticoagulation often entails a considerable reduction in quality of life. Furthermore, in the case of female patients, the possibility of pregnancy must be ruled out. In the context of endocarditis, it is our contention that treatment should be guided and inspired by principles regarding the avoidance of infection recurrence and valve functional outcomes. This is because redo surgery in the event of reinfection is particularly challenging and burdened by an augmented risk [5,6].

A significant study conducted in Germany demonstrated that CAHs treated with antibiotics retained antibacterial efficacy even after prolonged storage for over five years. The application of antibiotic combinations in the processing of CHs may exert a considerable effect on their anti-infective properties [165]. Particularly, the recurrent infection of prosthetic devices or prosthetic components, such as a mechanical prosthetic or stented bioprosthetic valve, presents a more formidable challenge and technical complexity than re-endocarditis in a prior CAH. In this context, there has been extensive reporting on the evidence base concerning the safety and effectiveness of CAH in comparison with conventional prostheses. This evidence has emerged from several observational studies [19,153,154,157,158,159,160,161,185,186]. A 10-year follow-up of patients who had undergone surgery for aortic valve endocarditis revealed that 2% of them experienced a relapse or recurrence of infection within the first year [152]. A review of the literature reveals that a recently published study demonstrated a reduced incidence of endocarditis in CAHs, even in cases with complex structural injuries to the cardiac structures [185].

In considering the long-term implications, the second question for the surgeon when selecting an allogenic tissue is the durability over time and the risk of a redo operation for SVD due to calcification of the CAH in comparison with conventional prostheses. In such circumstances, the requisite skill of the surgeon is considerable, given the technically demanding nature of the reintervention. Mortality rates have been reported in the literature as between 4 and 10%, with morbidity rates at 34% [187]. It is imperative to recognise that extensive demolition of adhesions is a crucial step in accessing the heart, given that a synthetic material was previously introduced. When compared with the reoperation for re-endocarditis on a prosthetic valve, the latter is more complex and carries a higher risk than the former, particularly in cases where a previously implanted homograft is involved. The presence of foreign material within the stent of a mechanical or biological prosthetic valve can result in a robust inflammatory reaction, potentially leading to the formation of stronger adhesions and complicating the surgical procedure. It is currently not possible to provide definitive responses based on conclusive immunological evidence regarding the degree of the proinflammatory immune response observed in allogenic grafts in comparison with xenografts.

The clinical experience with decellularized aortic homografts in children demonstrated favourable mid-term outcomes with regard to SVD. However, there is currently a paucity of evidence regarding the recurrence of IE [188]. While xenografts, such as biological prosthetic valves, are typically processed with chemical fixatives to prevent degeneration, they often contain a considerable amount of foreign material, including valve stents and suture skirts. This can potentially trigger an even more robust inflammatory response, leading to the formation of annular pannus, particularly when implanted with pledgeted sutures. In this context, an aggressive tissue reaction may prove to be a significant challenge. In order to achieve the adequate clearance of adherences, extensive surgical intervention may be necessary, which carries an inherent risk. Nevertheless, in contrast to prosthetic valves, CAHs are rarely affected by reinfection. The management of SVD in a homograft carries a relatively minor burden in comparison with redo surgery for an infected prosthetic valve. The utilisation of homografts and its deleterious effect on the immune system must be contextualized within the clinical scenario of endocarditis. Within this framework, the key tenets of treatment are the maintenance of good haemodynamic function and the avoidance of long-term anticoagulation. This is particularly relevant in the case of young patients. Additionally, there is a greater emphasis placed on the avoidance of redo surgery, which can favour the utilisation of homografts. This is especially pertinent when extensive endocarditis affects the aortic and mitral structures [14,19,22,153,154,163].

Despite the absence of international guideline recommendations for TAVR, the utilisation of sutureless aortic valve implantation in elderly patients presenting with comorbidities and PVE has the potential to offer substantial benefits in the field of valve replacement [5,6]. Nevertheless, the employment of TAVR and sutureless aortic valve replacement in the setting of SVD has the potential to significantly contribute to advancements in the field of valve replacement. These techniques can address complex injuries when traditional surgical approaches are not feasible [189,190]. In the event of significant calcification of the CAH, these procedures may be employed with a comparatively reduced risk profile in comparison with standard aortic valve replacement. The transcatheter aortic valve-in-valve procedure has provided substantial support in the treatment of SVD occurring in biological substitutes implanted in the aortic position. This consolidated technique was initially proposed by Kowert et al. [191] who reported survival rates of 86.0% and 77.4% after one and five years, respectively, following a redo operation involving a homograft. Furthermore, they identified transapical TAVR as a safe and feasible procedure, provided that the valve is not infected. Following the experience of Dainese et al. [192] who described the use of sutureless techniques in younger patients requiring reoperation for PVE and TAVR in older patients with promising results in reducing mortality and morbidity, other centres have adopted this approach [189,190,193,194,195]. Nevertheless, the prevalence and impact of a patient–prosthesis mismatch or of subclinical thrombosis (also known as hypoattenuated leaflet thickening, or HALT, and abnormal motion) remain undetermined due to the restricted number of RCT studies currently available and the lack of large-scale RCTs comparing haemodynamic outcomes with surgical outcomes [196].

It is recommended that the new generation of stented bioprosthetic valves for aortic valve disease, namely, the so-called “sutureless” (e.g., Perceval, Livanova) and “rapid-deployment” (Intuity, Edwards) prostheses, is given special consideration. Such prostheses represent an advancement over conventional stented bioprosthetic valve, offering a rapid implantation process. A special stent, comparable with that used in TAVR procedures, eliminates the need for sutures during implantation, either through a sutureless technique or a rapid deployment method requiring only three sutures. The use of these prostheses is contingent upon the integrity of the aortic annulus, which must be intact to ensure stability. Consequently, they were initially indicated for the treatment of aortic valve stenosis. Additionally, the presence of endocarditis was an exclusion criterion for their use [193]. Nevertheless, their capacity to markedly reduce surgical times represented a theoretical advantage for high-risk patients with severe disease and compromised haemodynamic conditions. The off-label use of the sutureless prosthesis as a valve substitute for patients affected by endocarditis has been documented in a limited number of cases with favourable outcomes [194]. A single report from Germany demonstrated the use of this prosthesis in a mitral position in a case of endocarditis where an adequate valvular substitute was not available [195]. To the best of our knowledge, there have been no reported instances of the Intuity valve being used in the treatment of endocarditis.

It is also important to consider the role of patient opinion in determining the choice of treatment, which can influence the surgeon’s ethical decision-making process. As Stulak et al. [193] observed in the context of pulmonary autograft use in Ross operations, the potential for failure or the necessity for reoperations introduces ethical considerations when considering the use of biological derivatives. It is, therefore, pertinent to consider whether some patients would prefer to take the risk of the potential recurrence of infection, and whether they would be willing to accept the possibility of a vast and extensively radical debridement in order to do so. It is pertinent to inquire whether the surgeon is obliged to apprise the patient of the technical intricacies associated with the utilisation of a homograft, which may potentially present challenges during the surgical procedure. This information may be particularly crucial in cases where the patient is otherwise healthy or young. The age of the patient may also be a determining factor in the choice of prosthesis, even in the presence of complex valve endocarditis with a largely infected field. What are the relative risks of redo operations using a homograft versus a prosthesis? It is evident that the heart team discussion must take into account the patient’s preference and willingness. A highly invasive procedure may be perceived as intimidating by patients and healthcare professionals. It is, therefore, essential to provide comprehensive information regarding the specifics of the procedure, potential complications, and postoperative course. This enables patients to make informed decisions. The patient must be informed of the complexity of the disease and the potential necessity for extensive debridement in order to achieve favourable and stable results. A more expedient surgical procedure involving the implantation of a prosthetic valve in the context of a substantial infective involvement of the aortic tissues presents a highly unstable situation with an elevated risk of infection recurrence. It is our contention that the option of undergoing a minimal operation with the known potential for reinfection should be discouraged [176,197,198,199,200,201,202].

## 8. Prognostic Features

It has been demonstrated that the nonoperative management of infective endocarditis in cases where surgery is indicated carries an elevated risk of both in-hospital mortality and mortality at one year [203,204,205,206]. In. opposition to this, the use of medical treatment alone for PVE is associated with an increased risk of relapse, particularly in cases of PVE caused by *Enterococcus faecalis* [35,36,203] or *Staphylococcus aureus* [1,204,205].

Despite advances in medical treatment and surgical techniques, the mortality rate for PVE continues to exceed that of native infective endocarditis [149,206,207]. Conversely, survivors of both conditions exhibit comparable long-term survival rates [205,207]. After adjusting for potential biases in treatment selection and survival outcomes, the elevated rate of perioperative mortality among patients in the ICE-PCS (International Collaboration on Endocarditis Prospective Cohort Study) multicentric cohort led to a reassessment of the efficacy of surgical intervention in prosthetic valve endocarditis [14,20,21,129]. The ongoing challenge to interpret these datasets comes from leading institutions that offer lower-risk operative care [148]. Indeed, outcomes for surgery in patients presenting with PVE have significantly improved since these data were first collected [208]. Moreover, the handling of anatomical issues, such as abscesses, fistulas, and the necessity for intervalvular fibrosa reconstruction [14,19,209], continues to present a significant challenge, as evidenced by poorer perioperative outcomes [210].

To avoid early PVE, certain precautions must be taken during the implantation surgery. It is essential to ensure the prosthetic material is not contaminated and to take steps to prevent prosthetic seeding from causing bloodstream infections in the first few days after the operation [211,212,213]. Preoperative antibiotic prophylaxis is also of great importance and must be administered at the optimal time and with an appropriate antimicrobial spectrum [211]. The recommended course of action is the use of a first-generation cephalosporin (such as cefazolin) for patients undergoing surgery for conventional cardiac procedures [212]. It is advisable to consider including prophylaxis for E. faecalis regarding percutaneous aortic prostheses. Amoxicillin/clavulanic acid should be used, or it can be combined with glycopeptides, such as vancomycin or teicoplanin, in conjunction with cefazolin [35,36,213]. Strategies to prevent late PVE must be implemented. Measures should be taken to promote good oral hygiene and administer antibiotic prophylaxis prior to invasive operations on the dentition. Nevertheless, this approach has been questioned, as research suggests the risk of bacteraemia from dental procedures may be lower than thought. A recently conducted American study has indicated a notable temporal correlation between specific oral health treatments and hospital admissions for endocarditis in individuals with underlying heart conditions. The study employed a case-crossover methodology, which provides clear and compelling evidence [214]. The research team was also correct in noting that antibiotic prophylaxis has a beneficial effect in preventing IE admissions. It is, therefore, recommended that those at-risk patients who undertake this course of treatment do so [214].

The registry-based clinical study (Effectiveness of Antibiotic Prophylaxis of Infective Endocarditis for Invasive Dental Procedures in Patients with Prosthetic Heart Valves and/or History of Infective Endocarditis [PROPHETS]; NCT05613933), initiated in France, will definitively answer this question by overcoming the limitations associated with a traditional clinical trial.

New evidence and ongoing research have prompted a change in recommendations for antibiotic choice in patients who are allergic to penicillin. While aminopenicillins (amoxicillin or ampicillin) and cephalosporins (cefazolin, cephalexin, or ceftriaxone) remain the preferred pharmacological agents, it is no longer recommended to use clindamycin in penicillin-allergic patients. This is due to the increased risk of *Clostridium difficile* infection. The aforementioned medication has been substituted with either azithromycin/clarithromycin or doxycycline [214,215,216,217,218,219].

## 9. Conclusions

PVE is not a static disease entity. It is, in fact, a dynamic process that undergoes continual evolution. Given the 40% to 50% one-year mortality rate, there is an urgent requirement for advances in the prevention, diagnosis, and management of PVE. As is the case with native valve IE, there is no question that clinical trials must be conducted across the full spectrum of this life-threatening infection. Despite the significant contributions from the heart team, the current evaluation of ongoing and planned investigations reveals the need for continued improvements in PVE prevention, diagnosis, and management.

Translating advances in materials science into prosthetic devices with reduced susceptibility to bacterial adhesion would be revolutionary. It may be beneficial to further investigate innovative material technologies that prevent interaction between bacteria and prosthetic surfaces (also known as low-fouling coats) or contain long-lasting bactericidal coatings. Despite previous research showing promise, there is still a need for further development before these can be implemented in clinical practice [220,221,222].

Understanding the relative importance of dental procedures for patients with known cardiac risk factors would help direct the use of antibiotic prophylaxis. The utility of integrated diagnostic strategies utilising multimodality imaging is becoming more evident; however, further refinement is necessary based on evidence obtained from real-world clinical populations. Surgical intervention is becoming a more prominent aspect, yet the considerable interinstitutional variation in outcomes highlights a necessity for more concentrated management in larger valve centres of excellence. It is crucial to enhance the quality and scope of the evidence base through additional RCTs. Currently, there are only six RCTs in IE that are listed as recruiting, as evidenced by the literature at the initial drafting of this document. It is notable that none of the RCTs included patients with the objective of developing a vaccine against staphylococcus aureus IE, following the unsuccessful outcome of the previous trial [223,224,225].

The design of IE may present certain difficulties, but the trials themselves are eminently achievable. Furthermore, they could be used to assess novel antibiotic strategies, as well as indications for surgery and the optimal timing of surgery. The ESC and AHA, in conjunction with the surgical societies, are uniquely suited to oversee the design and coordination of such studies, which will require a multicentre, multinational approach and reliance on hard endpoints, including mortality, as opposed to composite endpoints. The present circumstances present a unique opportunity to address the current challenges in IE and to identify solutions.

## Figures and Tables

**Table 1:**
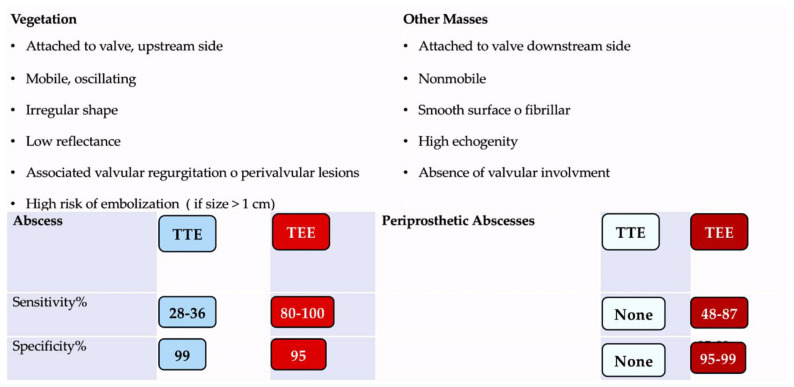
Sentitivity and specificity of echocardiography in detecting abscesses.

**Table 2:** A comparative analysis of the various diagnostic criteria employed in the diagnosis of infective endocarditis. The amendments to the Duke 2000 criteria are indicated by blue bold font, while the 2023 ESC is highlighted in dark red, the 2023 ESC is shown in violet, and the 2023 Duke/ISCVID is indicated by red bold font [7,8,11].

**2000 Modified DUKE Criteria**	**2015 and 2023 European Society of Cardiology (ESC) Criteria**	**2023 DUKE/International Society for Cardiovascular Infectious Diseases (ISCVID) Criteria**
**MAJOR CRITERIA**	**MAJOR CRITERIA**	**MAJOR CRITERIA**
**Microbiologic Major Criteria**	**Microbiologic Major Criteria**	**Microbiologic Major Criteria**
**Blood Cultures**	**Blood Cultures**	**Blood Cultures**
Typical microorganisms consistent with IE from two separate blood cultures: *Viridans streptococci, Streptococcus bovis, HACEK group, Staphylococcus aureus, or community-acquired enterococci, in the absence of a primary focus*	Typical microorganisms consistent with IE from two separate blood cultures: *Viridans streptococci, Streptococcus bovis, HACEK group, Staphylococcus aureus, or* ***Enterococcus faecalis***	Microorganisms that commonly cause IE * isolated from two or more separate blood culture sets. * *Staphylococcus aureus*, ***Staphylococcus lugdunensis*, *Enterococcus faecalis***, *streptococci of the following groups: S. mitis/oralis* (including *S. peroris and excluding S. pneumoniae*), *S. bovis/equinus* (including *S. gallolyticus* and *S. infantarius*), *S. sanguinis/parasanguinis*, *S. salivarius* (including *S. vestibularis*), *S. mutans* (including *S. sobrinus*), *S. gordonii*, *S. cristatus/sinensis*, ***Granulicatella* spp., *Abiotrophia defectiva*, *Gemella* spp.,** *HACEK* group organisms (*Haemophilus* species, *Aggregatibacter actinomycetemcomitans*, *Cardiobacterium hominis*, *Eikenella corrodens*, *Kingella kingae*)
Microorganisms consistent with IE from persistently positive blood cultures, defined as follows: At least 2 positive cultures of blood samples drawn 112 h apart or all of 3 or a majority of >4 separate cultures of blood (with first and last sample drawn at least 1 h apart)	Microorganisms consistent with IE from persistently positive blood cultures, defined as follows: At least 2 positive cultures of blood samples drawn > 12 h apart or all of 3 or a majority of >4 separate cultures of blood (with first and last sample drawn at least 1 h apart)	**Infectious agents that infrequently or intermittently induce IE, identified from three or more discrete blood culture samples.**
**2000 Modified DUKE Criteria**	**2015 and 2013 European Society of Cardiology (ESC) Criteria**	**2023 DUKE/International Society for Cardiovascular Infectious Diseases (ISCVID) Criteria**
**MAJOR CRITERIA**	**MAJOR CRITERIA**	**MAJOR CRITERIA**
**Laboratory Tests**	**Laboratory Tests**	**Laboratory Tests**
**A confirmed positive blood culture for Coxiella burnetii or an antiphase I IgG antibody titre of >1:800 is the definitive indicator.**	**A confirmed positive blood culture for Coxiella burnetii or an antiphase I IgG antibody titre of >1:800 is the definitive indicator**	**A positive PCR or other nucleic-acid-based technique (amplicon (16S or 18S) or shotgun (metagenomic) sequencing) for *Coxiella burnetii*, *Bartonella* species, or Tropheryma whipplei from blood. The patient must have a *Coxiella burnetii* antiphase I IgG antibody titre of >1:800, or the bacteria must be isolated from a single blood culture. The definitive test is an indirect immunofluorescence assay (IFA) for the detection of IgM and IgG antibodies to Bartonella henselae or Bartonella quintana with an IgG titre of >1:800.**
**Imaging Major Criteria**	**Imaging Major Criteria**	**Imaging Major Criteria**
The echocardiogram is positive for IE. This is defined as follows: The following are indicative of IE: - An oscillating intracardiac mass on the valve or supporting structures, in the path of regurgitant jets, or on implanted material in the absence of an alternative anatomic explanation; - An abscess; - A new partial dehiscence of prosthetic valve. - New valvular regurgitation (defined as worsening or alteration of a pre-existing murmur).	The echocardiogram shows a positive result for IE. The following are present: Vegetation; Abscess, pseudoaneurysm, or intracardiac fistula; Valvular perforation or aneurysm; New partial dehiscence of prosthetic valve. The presence of aberrant activity at the site of prosthetic valve implantation is unequivocally identified by [18F]FDG PET/CT (irrespective of the interval from surgery) or radiolabelled leukocytes SPECT/CT. Definite paravalvular lesions by cardiac CT.	Echocardiography **and/or Cardiac CT ** showing: Vegetation; Valvular/leaflet perforation; Valvular/leaflet aneurysm; Abscess, pseudoaneurysm, or intracardiac fistula. New partial dehiscence of prosthetic valve as compared with previous imaging. **The echocardiography shows significantly worse valvular regurgitation than the previous imaging. Worsening or changing of pre-existing regurgitation is not sufficient.** **[18F]FDG PET/CT metabolic activity involving a native or prosthetic valve, ascending aortic graft (with concomitant evidence of valve involvement), cardiac device leads, or other intracardiac prosthetic material, detected at least 3 months after prosthetic valve surgical implantation, is abnormal.**

Abbreviations: ESC, European Society of Cardiology criteria; ISCVID, International Society for Cardiovascular Infectious Diseases.

**Table 3:** The table depicts data from Pericà S et al. [9]. The term “highly difficult-to-treat microorganisms” is used to describe those requiring intravenous antibiotic combinations that cannot be administered by means of OPAT or that require the strict monitoring of drug levels either in blood or in other fluids, due to their potential toxicity or narrow therapeutic index (e.g., methicillin-resistant *Staphylococcus aureus*). Additionally, there are strains of *Staphylococcus aureus* that are resistant to both penicillin and vancomycin, as well as enterococci that are resistant to vancomycin and other drugs, such as daptomycin and linezolid. There are also multidrug-resistant and extensively drug-resistant Gram-negative rods, as well as highly penicillin-resistant viridans group streptococci. Finally, there are fungal strains that are resistant to penicillin, with the exception of those belonging to the *Candida* species. Abbreviations: OPAT; outpatient parenteral antibiotic treatment. Other abbreviations are in the other tables and figures.

GAMES Investigators’ Criteria for Using OPAT in Prosthetic Valve Endocarditis Patients		
Recommendation	Indications	Applications
A rapid transfer to OPAT is to be initiated at the 10-day mark following admission.	This indication covers all cases caused by viridans or *bovis* (*gallolyticus*) group streptococci or *Enterococcus faecalis*, provided that the patient is not undergoing cardiac surgery.	The blood cultures taken at 72 h yielded negative results. There were no severe clinical complications, no anticoagulation issues, and a TEE ruled out severe aortic regurgitation and prosthetic dysfunction.
The transfer to OPAT is postponed for a minimum of three weeks after admission/surgery.	This indication applies to all cardiac surgery cases that do not fall into any of the following two categories: Those caused by microorganisms that are highly difficult to treat; Those that present with severe complications.	The blood cultures taken at 72 h yielded negative results. There were no severe clinical complications, no anticoagulation issues, and a TEE ruled out severe aortic regurgitation and prosthetic dysfunction; There are no severe sequelae or clinical complications; There is a need for frequent and/or complex treatments.

**Table 4:** A comparative analysis of the various surgical criteria employed in the diagnosis of infective endocarditis. The amendments to the Duke 2000 criteria are indicated by blue bold font, while the 2023 ESC is highlighted in dark red, the 2023 ESC is shown in violet, and the 2023 Duke/ISCVID is indicated by red bold font [7,8,11].

2000 Modified DUKE Criteria	2015 and 2013 European Society of Cardiology (ESC) Criteria	2023 DUKE/International Society for Cardiovascular Infectious Diseases (ISCVID) Criteria
**Surgical Major Criteria**	**Surgical Major Criteria**	**Surgical Major Criteria**
-	-	**Evidence of IE was documented by direct inspection during the course of cardiac surgery, without subsequent histologic or microbiologic confirmation.**
**Minor Criteria**	**Minor Criteria**	**Minor Criteria**
Predisposition: heart condition or drug use	Predisposition: heart condition or drug use	**Predisposition:** - **Previous history of IE;** - Prosthetic valve; - Previous valve repair; - Congenital heart disease; - More than mild regurgitation or stenosis of any aetiology; - **Endovascular CIED;** - Hypertrophic obstructive cardiomyopathy; - Injection drug use.
Fever, temperature > 38 °C	Fever, temperature > 38 °C	Fever, temperature > 38 °C
**Vascular manifestations**, major arterial emboli, septic pulmonary infarcts, mycotic aneurysms, intracranial haemorrhages, conjunctival haemorrhages, and Janeway lesions.	**Vascular manifestation ****(including those detected by imaging only): **major arterial emboli, septic pulmonary infarcts, infectious (mycotic) aneurysm, intracranial haemorrhage, conjunctival haemorrhages, and Janeway’s lesions.	**Vascular manifestation: **Clinical or radiological evidence of arterial emboli, septic pulmonary infarcts, **cerebral or splenic abscess, hematogenous spondylodiscitis**, mycotic aneurysm, intracranial haemorrhage, conjunctival haemorrhages, Janeway lesions, and **purulent purpura**.
**Microbiological evidence: **positive blood culture but does not meet a major criterion as noted above or serological evidence of active infection with organism consistent with IE.	**Microbiological evidence: **positive blood culture but does not meet a major criterion as noted above or serological evidence of active infection with organism consistent with IE.	**Microbiologic evidence**, falling short of a major criterion: (1) Positive blood cultures for an organism consistent with IE but not meeting the requirements for major criterion. **(2)** **Positive culture, PCR, or other nucleic-acid-based test (amplicon or shotgun sequencing, in situ.**
**Immunologic manifestation: **glomerulonephritis, Osler’s nodes, Roth’s spots, and rheumatoid factor.	**Immunologic manifestation: **glomerulonephritis, Osler’s nodes, Roth’s spots, and rheumatoid factor.	**Immunologic manifestation: **immune-complex-mediated glomerulonephritis, Osler’s nodes, Roth’s spots, and positive rheumatoid factor.

The amendments to the Duke 2000 criteria are indicated by blue bold font, while the 2023 ESC is highlighted in dark red, the 2023 ESC is shown in violet, and the 2023 Duke/ISCVID is indicated by red bold font [7,8,11].

**Table 5:** Infective endocarditis in TAVR studies.

First Author, Year (Ref. ϕ)	No. of TAVR-IE Patients	Microbiology	1-Yr Incidence of TAVR-IE	In-Hospital Mortality	1-Yr-Mortality
Leon et al. PARTNER B, 2010 NEJM [136]	2 (cohort of 179)	Not indicated	1.12% γ	Not indicated	100%
Smith et al. PARTNER A, 2011 NEJM [137]	3 (cohort of 344)	Not indicated	0.87% γ	Not indicated	33%
Aung et al., 2013 SJID [141]	4 (cohort of 132)	*Enterococci* (75%), oral streptococci (25%)	3.0%	0%	0%
Latib et al., 2014 JACC [142]	29 (cohort of 2572)	*Enterococci* (21%), CoNS (17%), *S. aureus* (14%), oral streptococci (3.4%)	0.89% γ	45%	Not indicated
Olsen et al., 2015 CCI 2015 [143]	18 (cohort of 509)	*Enterococci* (33%), *S. aureus* (17%), oral streptococci (17%), CoNS (11%)	3.1%	11%	Not indicated
Puls et al., 2013 Eurointervention [144]	5 (cohort of 180)	*Enterococcus* (40%), oral streptococci (20%), *S. aureus* (20%), *E. coli* (20%)	2.78%	40%	40%
Mangner et al., 2016 JACC [138]	55 (cohort of 1820)	*S. aureus* (38%), enterococci (31%), CoNS (9.1%), oral streptococci (3.6%)	2.25% γ	64%	75%
Amat-Santos et al., 2015 Circulation [139]	53 (cohort of 7944)	CoNS (24%), *Staphylococcus aureus* (21%), enterococci (21%), oral streptococci (5.7%)	0.5%	47%	66%
Raguiero et al., 2016 JAMA [30]	250 (cohort of 20,006)	*Enterococcus* (25%), *S. aureus* (24%), CoNS (17%)	1.1% per person-year	36%	66.7% (2-yr mortality)
* Butt et al., 2019 JACC [145]	TAVR cohort of 2632	Not indicated	2.3%	20.9%	Higher risk of death HR: 2.05; 95% CI: 1.82 to 2.32
del Val et al., 2022 CJC [140]	604 (cohort of 40,345)	Non-*S. aureus* (432) *S. aureus* (141)	Non-*S. aureus* 6.3 months vs. *S. aureus* 4.7 months	*S. aureus* group (47.8% vs. 26.9%)	*S. aureus* group (71.5% vs. 49.6%)
** Amat-Santos et al., 2015 JACC cardio int [146]	60 32 TAVRs, 28 TPVRs (28 publications)	*Enterococcus* (34.4%), *S. aureus* (29.4%),	Not indicated	34.4%	TPVR-PVE patients (75%) were managed surgically, and in-hospital mortality was 7.1%.

Abbreviations: CI, confidence interval; CoNS, coagulase-negative staphylococci; HR, hazard ratio IE, infective endocarditis; PARTNER, Placement of Aortic Transcatheter Valve; TAVR, transcatheter aortic valve replacement.; TPVR, transcatheter pulmonary valve replacement. * IE in patients undergoing TAVR vs. SAVR; ** Metanalysis; γ Calculated/estimated.
